# Mitochondrial metabolism and cancer therapeutic innovation

**DOI:** 10.1038/s41392-025-02311-x

**Published:** 2025-08-04

**Authors:** Hongxiang Du, Tianhan Xu, Sihui Yu, Sufang Wu, Jiawen Zhang

**Affiliations:** 1 Department of Obstetrics and Gynecology, Shanghai General Hospital, Shanghai Jiao Tong University School of Medicine, Shanghai, China; 2 Department of Obstetrics and Gynecology, Zhongshan Hospital, Fudan University, Shanghai, China; 3 Department of Obstetrics and Gynecology, Shanghai Tongji Hospital, Tongji University School of Medicine, Shanghai, China; 4 Reproductive Medicine Center, Department of Obstetrics and Gynecology, Shanghai General Hospital, Shanghai Jiao Tong University School of Medicine, Shanghai, China

**Keywords:** Cancer microenvironment, Tumour immunology

## Abstract

Mitochondria are dynamic organelles that are essential for cellular energy generation, metabolic regulation, and signal transduction. Their structural complexity enables adaptive responses to diverse physiological demands. In cancer, mitochondria orchestrate multiple cellular processes critical to tumor development. Metabolic reprogramming enables cancer cells to exploit aerobic glycolysis, glutamine metabolism, and lipid alterations, supporting uncontrolled growth, survival, and treatment resistance. Genetic and epigenetic alterations in mitochondrial and nuclear DNA disrupt oxidative phosphorylation, tricarboxylic acid cycle dynamics, and redox homeostasis, driving oncogenic progression. Mitochondrial dysfunction in tumors is highly heterogeneous, influencing disease phenotypes and treatment responses across cancer types. Within the tumor microenvironment, mitochondria profoundly impact immune responses by modulating T-cell survival and function, macrophage polarization, NK cell cytotoxicity, and neutrophil activation. They also mediate stromal cell functions, particularly in cancer-associated fibroblasts and tumor endothelial cells. Although targeting mitochondrial function represents a promising therapeutic strategy, mitochondrial heterogeneity and adaptive resistance mechanisms complicate interventional approaches. Advances in mitochondrial genome editing, proteomics, and circulating mitochondrial DNA analysis have enhanced tumor diagnostic precision. This review synthesizes the developmental landscape of mitochondrial research in cancer, comprehensively summarizing mitochondrial structural dynamics, metabolic plasticity, signaling networks, and interactions with the tumor microenvironment. Finally, we discuss the translational challenges in developing effective mitochondria-based cancer interventions.

## Introduction

The intricate relationship between mitochondrial metabolism and cancer has emerged as a critical focal point in contemporary oncological research.^1^ In addition to their classical role as cellular power generators, mitochondria primarily generate adenosine triphosphate (ATP) through oxidative phosphorylation (OXPHOS). Their unique structural configuration, characterized by an outer membrane and highly folded inner membrane, facilitates critical electron transport chain (ETC) functions.^2^ This complex arrangement coordinates ATP synthase activity by leveraging the proton gradient generated during the process. Mitochondria serve as central regulators of apoptosis, particularly through the release of cytochrome c and other proapoptotic factors.^3^ Additionally, they maintain calcium homeostasis and coordinate reactive oxygen species (ROS) production, significantly influencing the cellular redox state.^4^ The mitochondrial genome encodes several proteins essential for cellular function. Through dynamic behaviors, including fission, fusion, and mitophagy, mitochondria rapidly adapt to cellular demands and environmental stressors.^5^

In the context of cancer, mitochondria orchestrate multiple cellular processes critical to tumor development. Cancer cells exhibit distinctive metabolic reprogramming, a cellular adaptation that rapidly rewires metabolic networks to support uncontrolled cell growth and survival, exemplified by the Warburg effect, which accelerates ATP generation and biosynthesis.^6^ This metabolic transformation is typically driven by oncogenic pathways, including the PI3K/Akt/mTOR and c-Myc pathways.^7^ Despite enhanced glycolysis, functional mitochondria remain crucial through multiple mechanisms. They regulate tricarboxylic acid (TCA) cycle intermediates during biosynthesis, maintain redox balance through glutamine metabolism, and coordinate lipid metabolism for energy production.^8^ Mitochondrial ROS (mitoROS) function as critical signaling molecules, promoting proliferation, angiogenesis, and immune evasion through pathways such as the NF-κB, MAPK, and PI3K/Akt pathways. Notably, mitochondrial DNA (mtDNA) mutations frequently occur in tumors, impacting OXPHOS efficiency and driving cancer progression.^9^

Research into mitochondrial metabolism has significantly enhanced our understanding of cancer biology and revealed potential innovative therapeutic targets. A comprehensive investigation of mitochondrial structural complexity, dynamic behaviors, roles in programmed cell death, and interactions with the tumor microenvironment (TME) will provide critical insights for the development of targeted cancer therapies.

This review commences with a historical perspective on mitochondrial research in oncology, followed by an in-depth exploration of mitochondrial structure and fundamental functions. Subsequent sections will critically examine the metabolic adaptations and stress responses of mitochondria in cancer, their participation in signaling networks, and their modulation of the TME. Finally, we assess how mitochondrial function influences therapeutic resistance, outline contemporary mitochondrial-targeted therapeutic strategies, including ongoing clinical trials, and conclude with a forward-looking perspective on future research directions and challenges.

### Historical perspective of mitochondrial research in cancer

The evolution of mitochondrial research, from basic cellular observations to complex molecular mechanisms, represents a cornerstone in understanding cancer biology. The study of mitochondria in cancer biology represents one of medicine’s most significant scientific journeys, encompassing over a century of discoveries and innovations.

The foundations of cancer mitochondrial research trace back to the 1920s, when Otto Warburg discovered a distinctive metabolic phenomenon in cancer cells. He reported that cancer cells preferentially rely on glycolysis for energy production, even under aerobic conditions, a process now known as the “Warburg effect”.^6^ This observation marked the initial recognition of mitochondrial functional alterations in cancer metabolism and sparked early investigations into the role of organelles in tumorigenesis.

The 1940s brought advances in electron microscopy, providing the first high-resolution images of mitochondria. These images revealed structural changes in cancer cells and offered early evidence linking mitochondrial abnormalities to tumor progression.^10^ Concurrent efforts to isolate mitochondria intensified during the 1940s and 1950s, culminating in the development of differential centrifugation techniques by pioneering cell biologists of that era.^11–13^ Their pioneering methods enabled functional mitochondrial isolation, which is crucial for identifying the respiratory chain and OXPHOS as core cellular energy generators. These achievements earned them the 1974 Nobel Prize in Physiology or Medicine. During this period, studies on mitochondrial OXPHOS efficiency in tumor cells provided crucial insights into cancer cell energy metabolism.^14^

The 1960s witnessed major advances in mitochondrial biochemistry, including the identification of essential enzymes for the TCA cycle and respiratory chain complexes. Key findings revealed that certain TCA cycle enzymes, such as succinate dehydrogenase (SDH) and fumarate hydratase (FH), act as tumor suppressors, establishing direct links between mitochondrial dysfunction and tumorigenesis.^15^

The discovery of mtDNA distinct from nuclear DNA in 1960s marked a pivotal moment.^16^ This breakthrough laid the foundation for subsequent genetic studies, culminating in Anderson et al.’s sequencing of the human mitochondrial genome in 1981, a revolutionary achievement enabling comprehensive studies of mtDNA mutations.^17^ By the late 1990s, researchers identified mtDNA alterations in various cancers, including colorectal and breast cancers, establishing crucial connections between mitochondrial genetics and tumorigenesis.^18^

The critical role of mitochondria in intrinsic apoptosis or programmed cell death became evident with the identification of cytochrome c release as a key apoptotic trigger,^19^ highlighting their central role in regulating cell death. The discovery of B-cell lymphoma 2 (BCL2) family proteins further emphasized the importance of mitochondria in controlling apoptosis, linking these organelles to cancer cell survival and therapy resistance.^20^ In the 1990s, ROS emerged as key mediators of cancer mitochondrial signaling. Studies have shown that mitochondrial dysfunction often leads to excessive ROS production, resulting in genomic instability, cancer progression, and metastasis.^21^

Technological advances have enabled new breakthroughs in understanding the roles of mitochondria in cancer biology.^22^ In 1997, Ichas et al. reported that mitochondria are excitable organelles capable of generating and conducting electrical and calcium signals.^23^ Live-cell imaging now enables real-time observation of mitochondrial dynamics, including fusion, fission, and mitophagy, and their impact on cancer progression.^4,24^ In 2019, Momcilovic et al. achieved in vivo imaging of cancer cell mitochondrial membrane potential via the voltage-sensitive positron emission tomography (PET) tracer 18F-benzyl triphenyl phosphonium (18F-BnTP).^25^ The integration of mitochondrial research with multi-omics technologies, including genomics, proteomics, and metabolomics, has provided unprecedented insights into tumor heterogeneity and microenvironmental adaptation.^26^ In 2023, Han et al. pioneered the mapping of the spatial distribution of cancer cell mitochondrial networks by integrating PET imaging, respirometry, and 3D scanning electron microscopy.^27^ In 2024, Kotrys et al. revealed dynamic patterns of mtDNA heterogeneity at single-cell resolution via single-cell combinatorial indexing leveraged to interrogate targeted expression (SCI-LITE), a novel high-throughput sequencing method combined with precise mtDNA base editing.^28^

These advances have accelerated the development of innovative therapeutic strategies. New approaches include drugs that inhibit OXPHOS, induce oxidative stress, or disrupt the mitochondrial membrane potential to selectively eliminate cancer cells.^29^ The discovery of small molecules that modulate mitochondrial respiratory chain activity has further expanded the scope of mitochondrion-targeted cancer therapies.^30^ In recent years, the emergence of mitochondrial dynamics inhibitors, immunometabolic modulators, and mitochondria-specific drug delivery systems has occurred.^31^ These advances have established foundations for personalized cancer treatment, highlighting mitochondrial quality control and metabolic vulnerabilities as promising therapeutic targets.

**Fig. 1:**
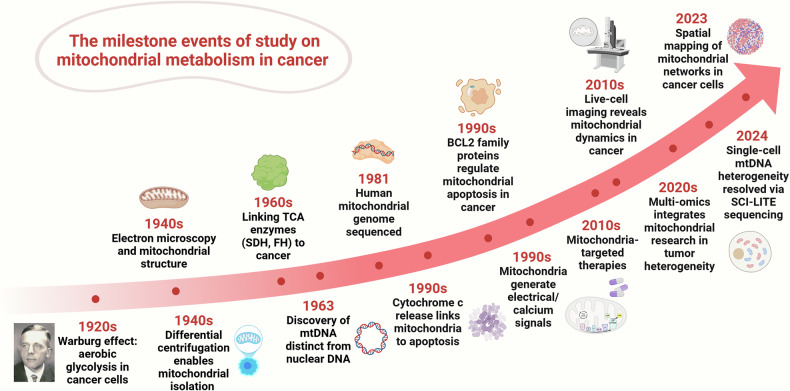
Milestone events in the study of mitochondrial metabolism in cancer. From the Warburg effect (1920s) to recent innovations such as spatial mitochondrial network mapping (2023) and single-cell mtDNA sequencing (2024), this timeline traces key discoveries in mitochondrial cancer research. Major breakthroughs include investigations of mitochondrial structural anomalies, tumor-suppressive metabolic enzymes, apoptosis regulation, and multi-omics integration. These findings have deepened our understanding of the roles of mitochondria in cancer metabolism, heterogeneity, and therapeutic targeting. SCI-LITE single-cell mitochondrial DNA lineage tracing and editing, mtDNA mitochondrial DNA, TCA cycle tricarboxylic acid cycle, BCL B-cell lymphoma 2. This figure was created with BioRender (https://biorender.com/)

As we continue to unravel the complexity of cancer mitochondrial function, these historical milestones demonstrate the central role of mitochondria in carcinogenesis and highlight the potential of mitochondria-based therapeutic innovations. Figure 1 summarizes the key advances in cancer mitochondrial research, illustrating the journey from fundamental discoveries to modern therapeutic progress.

## Mitochondrial structure and basic functions

**Fig. 2:**
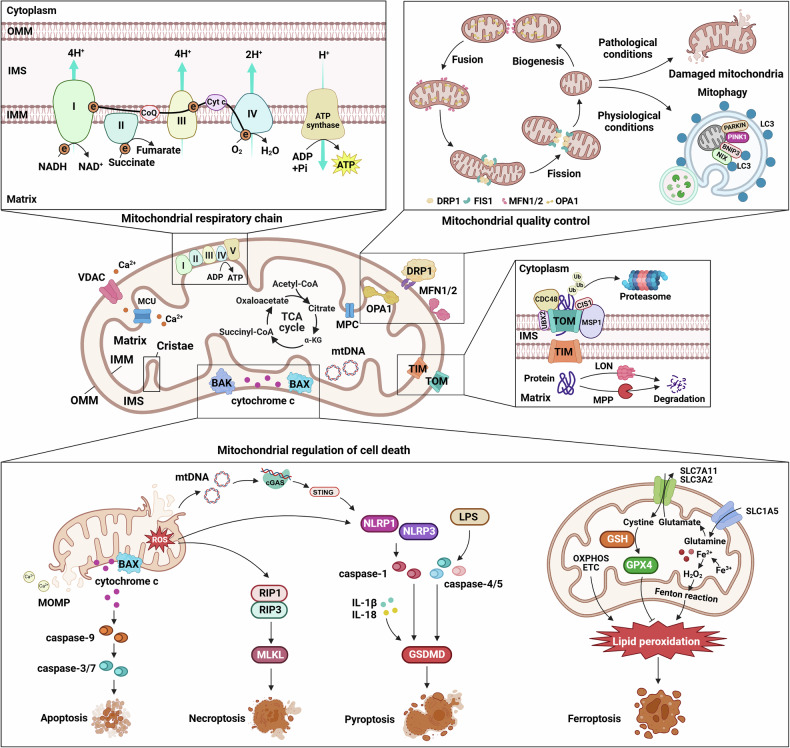
Comprehensive overview of mitochondrial functions, regulation, and cell death pathways. Mitochondria are complex cellular organelles with intricate architectures involving outer and inner membranes, cristae, and critical proteins that facilitate energy production and cellular regulation. The mitochondrial respiratory chain drives ATP synthesis through electron transfer and proton pumping, whereas quality control mechanisms such as fusion, fission, and mitophagy maintain cellular health. Multiple cell death pathways, including apoptosis, necroptosis, pyroptosis, and ferroptosis, are regulated by mitochondrial processes, involving key proteins and signaling molecules that mediate cellular responses to stress and damage, ultimately ensuring precise control of cell survival and elimination. ATP adenosine triphosphate, BAX BCL-2-associated X protein, DRP1 dynamin-related protein 1, ETC electron transport chain, FADH₂ flavin adenine dinucleotide, FIS1 mitochondrial fission 1 protein, GPX4 glutathione peroxidase 4, GSDMD gasdermin D, IMM inner mitochondrial membrane, LON Lon protease, MFN1/2 mitofusin 1/2, MLKL mixed lineage kinase domain-like protein, MCU mitochondrial calcium uniporter, MPP mitochondrial processing peptidase, mtDNA mitochondrial DNA, NADH nicotinamide adenine dinucleotide, NLRP1/NLRP3 NOD-like receptor family pyrin domain-containing 1/3, OMM outer mitochondrial membrane, OXPHOS oxidative phosphorylation, RIP1/RIP3 receptor-interacting serine/threonine-protein kinase 1/3, SLC7A11 solute carrier family 7 member 11, TCA cycle tricarboxylic acid cycle, TIM translocase of the inner membrane, TOM translocase of the outer membrane, VDAC voltage-dependent anion channel. This figure was created with BioRender (https://biorender.com/)

Mitochondria, commonly known as the “powerhouses of the cell”, are the major sites of ATP production through OXPHOS. In addition to energy production, they regulate crucial cellular processes, including signaling pathways, calcium homeostasis, and apoptosis. The complex mitochondrial architecture supports its diverse functions, making it indispensable for cell survival, growth, and adaptation (Fig. 2).^1,5^ In cancer cells, alterations in mitochondrial structure and function represent key features of tumor development and progression, influencing metabolic reprogramming and survival capabilities.

### Structural organization and functions

#### Membrane organization

Mitochondria are dynamic organelles characterized by a double-membrane structure comprising outer and inner membranes separated by an intermembrane space (IMS).

The outer mitochondrial membrane (OMM) is relatively permeable to porin channels, allowing the passage of small molecules and ions between the cytosol and IMS.^32^ This permeability facilitates essential metabolite transport for mitochondrial functions, supporting substrate exchange for ATP production and other metabolic processes.^33^ During apoptosis, mitochondrial outer membrane permeabilization (MOMP) propagates as waves through the cytoplasm and is regulated by casein kinase II (CK2).^34^ The OMM harbors enzymes involved in lipid metabolism, contributing to organelle lipid homeostasis. In cancer cells, multiple OMM proteins are aberrantly expressed. TOMM20, TOMM34, and FUNDC2 are frequently upregulated, which is correlated with tumor proliferation, migration and invasion.^35^ Voltage-dependent anion channel 1 (VDAC1) regulates energy metabolism, calcium homeostasis, and apoptosis in tumorigenesis.^36^ Recent research identifies voltage-dependent anion channel 2 (VDAC2) as a crucial immune signaling-dependent checkpoint in tumors. VDAC2 deficiency leads to IFNγ-induced BAK hyperactivation and mitochondrial damage, promoting mitochondrial DNA release into the cytosol, thereby activating the cGAS-STING pathway and type I interferon responses.^37^

The inner mitochondrial membrane (IMM) maintains strict impermeability to ions and small molecules, a crucial feature for sustaining the proton gradient necessary for ATP synthesis. This impermeability enables the IMM to maintain the unique ionic environment essential for mitochondrial function.^38^ The IMM is extensively folded into structures called cristae, increasing the surface area available for ETC components. Cristae arrangement and density vary by cell type, with energy-demanding tissues such as cardiac muscle displaying dense cristae to optimize ATP generation.^39^ In cancer cells, multiple IMM proteins are dysregulated. Translocase of inner mitochondrial membrane 44 (TIMM44) is upregulated in bladder cancer, supporting tumor growth through maintenance of mitochondrial function and integrity.^40^ Similarly, Translocase of inner mitochondrial membrane 23 (TIMM23) overexpression promotes non-small cell lung cancer (NSCLC) growth by enhancing ATP production and membrane potential.^41^

The IMS plays vital roles in mitochondrial function, serving as a reservoir for proton pumping during electron transport. This proton gradient is essential for ATP synthesis and is generated by respiratory chain complexes in the IMM. The IMS serves crucial functions in calcium signaling and represents a highly protected compartment second only to the matrix.^42^ It contains critical proteins, including cytochrome c and Smac/DIABLO, whose release regulates apoptotic processes. The IMS also functions as a protein transport and folding hub, featuring the MIA40/ERV1-mediated disulfide relay system crucial for protein oxidative folding and assembly.^43^ Molecular chaperone complexes guide hydrophobic precursor proteins through this aqueous compartment. Recent discoveries have revealed that the IMS protein Skd3 is crucial for the disaggregation activities essential for human health.^44^ In cancer cells, elevated oxidative stress leads to H_2_O_2_ accumulation in the IMS, promoting tumor growth.^45^ Multiple IMS proteins are aberrantly expressed, with 4-hydroxyphenylpyruvate dioxygenase-like (HPDL) promoting mitochondrial energy metabolism in pancreatic cancer and SLP-2 regulating the ROS and extracellular signal-regulated kinase (ERK) pathways in papillary thyroid cancer.^46^

The mitochondrial matrix, enclosed by the inner membrane, has intense biochemical activity. It contains an array of enzymes that drive the TCA cycle and are responsible for the oxidative degradation of metabolic fuels and the generation of electron carriers such as nicotinamide adenine dinucleotide + hydrogen (NADH) and flavin adenine dinucleotide (FADH2).^47^ The matrix houses mtDNA, which encodes a limited set of genes essential for mitochondrial function, primarily those involved in OXPHOS. Additionally, the matrix contains two major proteases, LON and ClpXP, which are responsible for protein quality control,^48^ whereas molecular chaperones such as mtHsp70 are crucial for mitochondrial protein import and folding.^49^ The matrix contains copper ion-ligand (CuL) complexes that provide copper ions for cytochrome c oxidase and superoxide dismutase assembly.^50^ Furthermore, matrix-localized Src kinase regulates mitochondrial morphology, whereas changes in matrix pH and volume are correlated with various physiological and pathological processes.^51^ In cancer cells, matrix protein homeostasis is disrupted, and OXPHOS function is altered, which is characterized by altered ATP synthase activity and matrix pH dysregulation.^52^ Moreover, cancer cells exhibit elevated matrix calcium ion concentrations and ROS levels, along with significant alterations in matrix volume and density.^53^

#### Respiratory chain complexes

The ETC, located in the mitochondrial inner membrane, comprises a series of protein complexes (complexes I-IV) crucial for ATP production.

##### Complex I: NADH-ubiquinone oxidoreductase

Complex I, the largest membrane protein complex in the mitochondrial respiratory chain (~980 kDa), consists of 44 distinct subunits. All redox cofactors reside in the peripheral arm rather than the membrane domain.^54^ Its core function involves transferring two electrons from NADH to ubiquinone while pumping four protons from the matrix to the IMS. This process couples electron transfer with proton pumping through long-range conformational changes, making it a key enzymatic complex for cellular energy metabolism and ROS generation. This mechanism is essential for establishing the proton gradient later used in ATP synthesis.^54^ Dysfunction of Complex I is associated with various neurological disorders. While complex I activation enhances oxidative phosphorylation through glutamine metabolism, promoting tumor progression in bladder cancer,^55^ reduced complex I activity in colorectal cancer contributes to chemoresistance by altering mitochondrial function,^56^ indicating complex and diverse roles across different tumor types.

##### Complex II: succinate-ubiquinone oxidoreductase

Complex II, the smallest membrane protein complex (124 kDa) in the respiratory chain, has a flower-like structure comprising four subunits.^57^ SDHA and SDHB form the hydrophilic head protruding into the matrix, whereas SDHC and SDHD form the hydrophobic stem anchored in the inner membrane. It contains flavin adenine dinucleotide (FAD) cofactors, iron-sulfur clusters, and ubiquinone binding sites, and its stability and activity are dependent on cardiolipin. The assembly intermediate structure shows that the FrdA subunit is crosslinked with its assembly factor SdhE, potentially forming a channel to the active site.^58^ Complex II catalyzes succinate oxidation to fumarate, which transfers electrons to ubiquinone without participating in proton transport, distinguishing it from other respiratory complexes.^59^ Despite not pumping protons, it plays crucial roles in maintaining electron flow and linking aerobic respiration with the energy-producing TCA cycle and steroid metabolism regulation.^60^ In cancer cells, complex II activation via METTL1-mediated m7G tRNA modification and SDHAF4 upregulation drives mitochondrial OXPHOS and gastric cancer progression,^61^ whereas complex II activity is downregulated by evolutionarily conserved selenoprotein O (SELENOO)-mediated AMPylation of SDHA, promoting melanoma metastasis through modulation of oxidative stress responses.^62^

##### Complex III: ubiquinol-cytochrome c oxidoreductase

Complex III is a dimeric cytochrome bc1 complex containing two major electron transfer sites: the Qo site and the ubiquinone binding site within the IMM. It contains multiple redox centers, including cytochrome b, cytochrome c1, and Rieske iron-sulfur proteins. The superoxide anions released into the IMS are metabolized by Cu,Zn-superoxide dismutase (SOD1), whereas the matrix-side superoxide is processed by Mn-SOD (SOD2). Complex III employs the Q-cycle mechanism to facilitate electron transfer from reduced ubiquinone to cytochrome c.^63^ This process involves a bifurcated electron transfer pathway, ensuring efficient electron flow while coupling transfer to proton translocation across membranes.^64^ Furthermore, Complex III forms supercomplexes with Complexes I and IV, an assembly mediated by respiratory chain supercomplex factors (RCFs) 1 and 2, whose stability is dependent on the presence of phosphatidylcholine. Complex III dysfunction or mutations can lead to impaired electron transfer and reduced ATP synthesis, which are associated with various mitochondrial diseases and metabolic disorders.^65^ In cancer, multiple complex III subunits show distinctive roles: UQCRC1 has oncogenic effects on pancreatic cancer,^66^ UQCRC2 overexpression is correlated with tumor progression and poor prognosis in colorectal cancer,^67^ whereas UQCRH acts as a tumor suppressor in clear cell renal cell carcinoma (ccRCC).^68^

##### Complex IV: cytochrome c oxidase

Complex IV, the terminal enzyme of the mitochondrial ETC, functions as a dimer composed of 13 subunits. The three largest subunits (COX I, II, and III), encoded by the mitochondrial genome, form the functional core, whereas the remaining ten subunits are encoded in the nucleus.^63^ It contains crucial proton channels, including the K-channel (transporting two protons to the catalytic site) and D-channel (composed of water molecules and conserved polar and protonable residues, including Glu242 and Asp91).^69^ Complex IV primarily transfers electrons from cytochrome c to molecular oxygen, forming water. This electron transfer couples with proton pumping across the inner membrane, significantly enhancing the proton electrochemical gradient required for ATP synthesis.^70^ Its regulation is crucial for maintaining efficient cellular respiration by optimizing ATP production while minimizing ROS generation.^63^ Complex IV dysregulation in cancer promotes tumor progression through subunit-specific mechanisms. COX4I2 functions as a hypoxia-associated gene driving epithelial-mesenchymal transition (EMT) and angiogenesis in colorectal cancer.^71^ COX6B2 enhances oxidative phosphorylation in pancreatic cancer.^72^ Moreover, copper depletion strategies targeting complex IV assembly show therapeutic potential by hindering mitochondrial metabolism in tumors.^73^

##### Complex V: ATP synthase

Complex V, a crucial enzyme in the mitochondrial ETC, is a multisubunit membrane-associated protein complex comprising two major subcomplexes: the hydrophilic F1 portion (responsible for ATP synthesis) and the hydrophobic Fo portion (responsible for proton transport).^74^ Human ATP synthase consists of 29 polypeptide chains from 18 subunits, with the F1 head region containing α3β3 subunits and the membrane Fo region including the c8 ring, ATP6 (or a), ATP8 (or A6L), e, f, g, DAPIT, and 6.8 PL subunits.^75^ Complex V synthesizes ATP from ADP and inorganic phosphate. This process depends on the proton motive force generated by preceding complexes, driving the rotation of the Fo and F1 subunits of ATP synthase.^76^ This rotary motion facilitates ATP production and release, demonstrating remarkable evolutionary adaptation for optimized energy generation.^77^ ATP synthase subunit regulation exhibits dual roles in cancer: ATP5F1D downregulation induces pyroptosis via mtROS/NLRP3/GSDMD pathway to inhibit endometrial cancer progression,^78^ whereas ACK1-mediated phosphorylation of ATP synthase F1 subunit alpha enhances prostate cancer survival while creating mitochondrial vulnerabilities.^79^

#### Ion transport

Mitochondria play crucial roles in maintaining the homeostasis of cellular ions, particularly calcium, potassium, and sodium, thereby influencing intracellular signaling and metabolic processes.

##### Calcium (Ca²⁺)

Mitochondrial calcium uptake is a critical process that regulates various metabolic pathways and intracellular signaling cascades. This uptake influences ATP synthesis, as calcium serves as a cofactor for mitochondrial dehydrogenases in the TCA cycle. Mitochondria buffer intracellular calcium levels, preventing cellular calcium overload and maintaining homeostasis.^80^ Additionally, mitochondrial calcium handling plays vital roles in regulating apoptosis, where excessive calcium can trigger cell death pathways.^4^ The mitochondrial calcium uniporter (MCU) is aberrantly expressed in various cancers. In colorectal cancer, increased MCU-mediated calcium uptake promotes mitochondrial biogenesis and tumor growth.^81^ MCU drives pancreatic cancer progression by enhancing mitochondrial Ca^2+^ uptake and regulating EMT-dependent metastasis.^82^ Furthermore, MCU regulates branched-chain amino acid catabolism via calcium-dependent PDH activity in fibrolamellar carcinoma.^83^

##### Potassium (K⁺)

Potassium ions are essential for maintaining the mitochondrial membrane potential and regulating cell volume. Mitochondria possess specific potassium transport mechanisms, including ATP-dependent potassium channels (mitoKATP) and calcium-activated potassium channels (mitoKCa).^84^ Proper potassium transport ensures optimal mitochondrial membrane potential function, which is crucial for ATP synthesis and overall metabolic regulation.^85^ Studies have revealed high expression of the mitochondrial potassium channel Kv1.3 in various cancer cells, where its inhibition leads to ROS-mediated tumor cell death.^86^ In malignancies, low expression or inhibitory regulation of mitochondrial potassium channels may contribute to drug tolerance.^87^

##### Sodium (Na⁺)

Mitochondria participate in sodium transport, an integral component of the cellular ion balance. Sodium ions typically exchange with calcium ions through the mitochondrial sodium-calcium exchanger (mNCX), regulating various signaling pathways and affecting mitochondrial function and overall cellular health.^88^ This exchange process is crucial for preventing calcium overload and maintaining cellular stability. In metastatic prostate cancer cells, increased sodium influx mediated by the Na^+^ leakage channel NALCN is correlated with invasiveness.^89^ In hepatocellular carcinoma cells, elevated intracellular sodium selectively kills cancer cells and leads to tumor reduction in mouse models.^90^ The expression of the mitochondrial sodium-calcium exchanger NCLX is reduced in colorectal cancer.^91^

##### Mitochondrial permeability transition pore (mPTP)

The mPTP, a nonselective channel in the mitochondrial inner membrane, allows the passage of molecules up to 1.5 kDa in size. Structurally, adenine nucleotide translocase (ANT) and mitochondrial F1FO-ATP synthase (particularly its dimers, monomers, or c-subunit rings) form the main molecular components of the pore, whereas cyclophilin D (CypD) facilitates pore formation.^74^ Multiple factors regulate mPTP opening, including calcium ions, ROS, and the membrane potential. The mPTP plays dual roles in cell survival and death, depending on the duration and extent of opening: transient openings participate in the physiological regulation of calcium homeostasis, bioenergetics, and redox balance, whereas sustained opening leads to mitochondrial swelling, outer membrane rupture, and subsequent apoptotic and necrotic death, which are important in various diseases.^92^ Studies have demonstrated that the mPTP opening status directly influences cancer cell survival and death. Changes in the expression of key proteins affect cancer progression through mPTP regulation. For example, CypD promotes transient mPTP opening, serving as a safe calcium efflux mechanism crucial for cancer cell survival.^93^ Notably, cancer cells and normal cells exhibit different mPTP characteristics, reflected in their responses to calcium ions and oxidative stress.

### Mitochondrial quality control

Mitochondrial function is influenced by both its intrinsic structure and its dynamic regulation.^94^ Mitochondria continuously undergo fusion and fission, crucial processes for maintaining their integrity and function, and population turnover. These dynamic behaviors must integrate with effective quality control mechanisms to ensure mitochondrial health and prevent cellular dysfunction.^94^ In cancer, disrupted mitochondrial dynamics lead to abnormal fusion or fission, affecting not only cancer cell energy metabolism but also tumor invasion and metastasis.

#### Mitochondrial biogenesis

Mitochondrial biogenesis represents the process of synthesizing new mitochondria driven by increased energy demands, involving the coordinated regulation of the mitochondrial and nuclear genomes.^95^ Mechanistically, peroxisome proliferator-activated receptor γ coactivator 1-α (PGC-1α) acts as a master regulator, controlling mitochondrial biogenesis by activating nuclear transcription factors (including NRF-1, NRF-2, and ERR-α). These transcription factors increase mitochondrial transcription factor A (TFAM) expression, which regulates mtDNA transcription and replication as the ultimate effector.^96^ Multiple signaling pathways regulate this process: AMP-activated protein kinase (AMPK) induces nuclear translocation of the transcription factor EB (TFEB) and increases PGC-1α and estrogen-related receptor alpha (ERRα) mRNA expression, leading to sequential lysosomal and mitochondrial biogenesis.^97^ Sirtuin 1 (SIRT1) mediates nuclear and mitochondrial gene transcription through PGC-1α activation, whereas Sirtuin 3 (SIRT3) promotes the expression of proteins involved in OXPHOS, the TCA cycle, and fatty acid oxidation.^98^ Functionally, mitochondrial biogenesis is closely related to cellular energy production, metabolic regulation, and quality control. It maintains the integrity and function of the mitochondrial population through balance among fusion, fission, and mitophagy. Additionally, it participates in calcium homeostasis, ROS generation, fatty acid β-oxidation, and amino acid metabolism regulation.^99^ Under pathological conditions, dysregulated mitochondrial biogenesis is associated with various diseases. In cancer, its abnormal enhancement is correlated with tumor invasion and metastasis.^100^

#### Mitochondrial fusion and fission

Mitochondrial fusion and fission are essential for maintaining mitochondrial morphology and function. Fusion is mediated by mitofusins 1 and 2 (MFN1/2) and OPA1, which coordinate outer and inner membrane fusion.^4^ This fusion facilitates mixing of mitochondrial contents, diluting damaged components and promoting genetic material exchange. Fusion becomes particularly crucial during stress or injury, helping maintain healthy mitochondrial populations and ensuring efficient ATP production.^4^ Conversely, mitochondrial fission is regulated by dynamin-related protein 1 (DRP1) and fission 1 protein (FIS1).^101^ Fission promotes the division of mitochondria into smaller entities, which are crucial for quality control, by segregating damaged or dysfunctional components for selective degradation.^102^ Additionally, fission is essential during mitosis, ensuring fair mitochondrial distribution among daughter cells. The delicate balance between fusion and fission is crucial for mitochondrial health.^94^ Studies have revealed widespread imbalances in mitochondrial dynamics across various tumors. Excessive fission promotes metabolic reprogramming in cancer cells.^103^ Meanwhile, this disruption also affects cancer cell invasiveness and cancer stem cell self-renewal capacity.^104^

#### Mitophagy

Mitophagy involves the selective degradation of damaged mitochondria to maintain their integrity. The PINK1/Parkin pathway plays a crucial role in this process. Under normal conditions, PINK1 is imported into healthy mitochondria for degradation. However, in damaged mitochondria, PINK1 accumulates on the outer membrane, where it recruits the E3 ubiquitin ligase Parkin.^105^ Parkin ubiquitinates several outer membrane proteins, marking these mitochondria for autophagosomal degradation.^106^ Mitophagy prevents the accumulation of dysfunctional mitochondria, which are crucial for cellular homeostasis. Factors such as elevated ROS levels or membrane potential loss can trigger mitophagy, allowing cells to respond to metabolic demands or injury.^107^ Mitophagy dysregulation is closely related to tumor development. In hepatocellular carcinoma, SLP-2 enhances tumor metastasis by promoting PINK1-mediated mitophagy.^108^ In colorectal cancer, ATF4-mediated PINK1/Parkin mitophagy pathway is activated to suppress lipid peroxidation during ferroptosis, suggesting that mitophagy-deficient tumors are more susceptible to ferroptosis-inducing therapies.^109^ In endometrial cancer, SIRT1 enhances mitophagy by deacetylating FOXO3, driving hormone resistance.^110^ Notably, aberrant expression of mitophagy receptors and adapters (including BNIP3, BNIP3L/NIX, and p62/SQSTM1) commonly occurs in tumors, providing important therapeutic targets.^111^

#### Protein quality control

Mitochondrial protein quality control works in concert with mitophagy to prevent harmful accumulation of misfolded or aggregated proteins within these organelles.^94^ Under both physiological and stress conditions, chaperone proteins such as Hsp60 and mtHsp70 ensure proper protein folding while being regulated by stress-responsive transcriptional programs, including heat shock factors.^112^ These chaperones are particularly crucial in cancer cells, which frequently experience significant oxidative stress and metabolic reprogramming that can destabilize the mitochondrial proteome. The proteolytic branch of this quality control system, characterized by key players such as the LON protease and ClpP, provides a second line of defense against protein aggregation.^113^ These proteases maintain mitochondrial protein integrity through selective degradation of irreversibly damaged peptides, thereby supporting mitochondrial function. Studies have revealed that LONP1 and ClpP can act synergistically to promote cancer cell survival through the regulation of mitochondrial protein homeostasis.^114^ LONP1 not only promotes colorectal cancer and skin tumor progression by regulating mitochondrial protein homeostasis and energy metabolism, but also influences cancer cell metabolic characteristics through interaction with FUN14 domain containing 1 (FUNDC1) on the mitochondrial inner membrane, modulating OXPHOS and ATP synthase activity.^115^ In glioblastoma, ankyrin repeat and zinc finger peptidyl TRNA hydrolase 1 (ANKZF1) knockdown suppresses tumors by promoting mitochondrial protein aggregation and affecting LONP1 function.^116^ Similarly, in pancreatic ductal adenocarcinoma, abnormal ClpP activation can inhibit tumor progression by disrupting mitochondrial protein homeostasis.^117^

#### mtDNA maintenance

Maintaining mtDNA integrity is crucial for normal mitochondrial function and cellular health. mtDNA replication occurs concurrently with mitochondrial biogenesis and involves specific mitochondrial factors, including DNA polymerase gamma (POLγ).^118^ Complementary repair pathways, such as base excision repair (BER) and double-strand break (DSB) repair, correct oxidative damage and ensure mtDNA fidelity.^119^ Mutations and deletions in mtDNA profoundly impact mitochondrial function and cellular metabolism, serving as functional modulators of cancer metabolism and tumor biology (Table 1). TFAM has emerged as a key regulator influencing tumor progression through its control of mtDNA replication, transcription, and maintenance. It also functions as an autophagy receptor, limiting inflammatory responses by binding cytosolic mtDNA.^120^ Notably, TFAM deletion in dendritic cells induces mitochondrial dysfunction and mtDNA cytosolic leakage, activating the cGAS-STING pathway and reversing the immunosuppressive TME.^121^ TNF receptor-associated protein 1 (TRAP1) interacts with mitochondrial quality and mtDNA copy number (mtDNA-CN) through the PGC-1α/TFAM signaling pathway, participating in the regulation of mitochondrial biogenesis in human CRC cells.^122^ Additionally, the regulation of mitochondrial dynamics is essential for maintaining mtDNA integrity and copy number, with its dysregulation affecting tumor cell metabolic reprogramming and invasive capabilities.^123^ A recent study revealed that human melanoma patients with >50% mtDNA mutations have ~2.5-fold higher response rates to checkpoint blockade.^124^ These observations suggest that mimicking mtDNA mutation effects might increase immunotherapy sensitivity in refractory cancers.

**Table 1:** Mitochondrial DNA and nuclear-encoded mitochondrial gene alterations in cancer

Mutant gene	Region	Type of cancer	Gene Function
MT-ND1, MT-ND2, MT-ND4, MT-ND5, MT-ND6	Complex I	Oral squamous cell carcinoma, testicular cancer, colorectal cancer, pancreatic cancer, multiple myeloma, cervical cancer, renal cell carcinoma	Electron transport chain dysfunction, ROS accumulation, metabolic reprogramming
MT-CYB	Complex III	Testicular cancer, ovarian cancer, colorectal cancer, clear cell renal cell carcinoma, neuroblastoma, breast cancer, oral cavity cancer	Dysregulated mitochondrial respiration, metabolic shifts, impaired mitochondrial dynamics
MT-CO1, MT-CO2, MT-CO3	Complex IV	Testicular cancer, brain gliomas, ovarian cancer, prostate cancer, endometrial cancer	Electron transport chain dysfunction, mitochondrial dysfunction
MT-ATP6, MT-ATP8	Complex V	Testicular cancer, ovarian cancer, brain gliomas, prostate cancer	Impaired ATP production, increased Warburg effect, reduced mitochondrial membrane potential
MT-RNR1, MT-RNR2	Ribosomal RNA	Testicular cancer, breast cancer, ovarian cancer, prostate cancer	Impaired mitochondrial protein synthesis, mitochondrial dysfunction, aberrant mitochondrial ribosome function
MT-TL1	Transfer RNA	Osteosarcoma	Mitochondrial translation defects, mitochondrial dysfunction, increased oxidative stress
SOD2	Inner mitochondrial membrane	Bladder cancer, breast cancer, cervical cancer, melanoma	Impaired ROS, increased oxidative stress
VDAC1	Mitochondrial outer membrane	Pancreatic cancer, breast cancer	Mitophagy dysfunction, abnormal calcium homeostasis
HSP10, HSP60, HSP70, HSP90	Matrix	Breast cancer, prostate carcinogenesis, stomach adenocarcinoma, colon adenocarcinoma	Increased oxidative stress, mitochondrial dysfunction

### Cell death pathways

Mitochondria serve as central regulators of various regulated cell death pathways, influencing cellular fate during stress and injury responses.^3^ Understanding these pathways is crucial for elucidating the underlying cancer mechanisms. The main forms of mitochondria-related cell death include apoptosis, ferroptosis, pyroptosis, and necroptosis.

#### Apoptosis

Apoptosis, or programmed cell death, represents a tightly regulated process that systematically eliminates damaged or unnecessary cells while minimizing inflammation. The intrinsic pathway, initiated by intracellular signals such as DNA damage and oxidative stress, heavily depends on mitochondrial integrity.^125^ A key event in this pathway is MOMP, which is mediated by BCL-2 family proteins. Pro-apoptotic proteins, including BAX and BAK, undergo conformational changes and oligomerize upon activation, leading to MOMP and cytochrome c release into the cytosol.^126^ Cytochrome c subsequently binds with Apaf-1 and dATP, forming the apoptosome, which recruits and activates initiator caspase-9. Activated caspase-9 then activates effector caspases, such as caspase-3 and caspase-7, resulting in the degradation of cellular substrates such as nuclear lamins and poly (ADP-ribose) polymerase (PARP).^127^ The extrinsic pathway is initiated through cell surface death receptors (e.g., Fas or TRAIL receptors) via extracellular signals. This receptor-ligand interaction activates caspase-8, which either directly initiates apoptosis or cleaves Bid into the truncated form (tBid).^128^ Recent studies have revealed that BAK protein tandem peptides (α4-α5, α5-α6, or α6-α7/8) can localize to mitochondria and permeabilize liposomes containing mitochondrial outer membrane lipids, inducing MOMP even without other BAK protein regions.^126^ Additionally, minority MOMP (miMOMP) in a subset of mitochondria characterize cellular senescence, requiring BAX and BAK macropores and releasing mtDNA into the cytosol. Under apoptotic conditions, significant enrichment of unsaturated lipids near BAK and BAX occurs. Moreover, fatty acid desaturase 2 (FADS2) enhances both apoptotic sensitivity and downstream cGAS/STING pathway activation following mtDNA release.^129^

#### Ferroptosis

Ferroptosis emerged as a novel form of regulated cell death in the 2010s. Unlike apoptosis, ferroptosis depends on iron and lipid peroxide accumulation. This process is initiated under conditions leading to uncontrolled ROS accumulation, promoting polyunsaturated fatty acid peroxidation in cell membranes.^130^ Free iron catalyzes these oxidative reactions, ultimately causing lipid peroxidation and cell death. Antioxidant defense systems, particularly glutathione (GSH) and its enzyme glutathione peroxidase 4 (GPX4), play crucial roles in ferroptosis regulation.^131^ GPX4 reduces lipid hydroperoxides to nontoxic alcohols; its depletion elevates lipid peroxides, triggering ferroptosis. As ROS sources and lipid metabolism participants, mitochondria are closely associated with ferroptosis, emphasizing the necessity of the prevention of oxidative stress and cell death in the context of mitochondrial health.^125^

Recent research revealed that OPA1, a mitochondrial dynamics-like GTPase, sensitizes cells to ferroptosis by maintaining mitochondrial homeostasis and function, facilitating mitochondrial lipid-reactive ROS generation and suppressing the ATF4-mediated integrated stress response.^132^ Additionally, diacyl polyunsaturated phosphatidylcholines (PC-PUFA2s) initiate lipid peroxidation through interactions with the mitochondrial ETC, which is correlated with cancer cell ferroptosis sensitivity.^133^ Mitochondrial outer membrane-anchored cGAS binds DRP1 to promote its aggregation. Without cGAS or DRP1 aggregation, mitoROS accumulation and ferroptosis increase, inhibiting tumor growth.^134^

#### Pyroptosis

Pyroptosis is an inflammatory form of cell death characterized by the activation of inflammatory caspases (caspase-1, caspase-4, and caspase-5), leading to cell lysis and the release of proinflammatory cytokines. Pattern recognition receptors (PRRs), particularly NOD-like receptors (NLRs), initiate this process by recognizing pathogens or danger signals, resulting in inflammasome formation.^135^ Inflammasomes activate caspase-1, processing and releasing the cytokines IL-1β and IL-18 and cleaving gasdermin D (GSDMD).^136^ Cleaved GSDMD forms pores in plasma membranes, causing osmotic swelling and lysis. MitoROS generation can activate the NLR family pyrin domain containing 3 (NLRP3) inflammasome, inducing pyroptosis,^137^ or trigger GSDME-dependent pyroptosis via the ROS/JNK/Bax pathway.^138^ Mitochondrial calcium overload promotes pyroptosis through calcium-dependent protease activation.^139^ Mitochondrial quality control plays crucial roles in pyroptosis regulation, where damaged mitochondrial removal (mitophagy) can suppress excessive pyroptotic responses.^137^ In cancer biology, the E3 ubiquitin ligase PJA1 promotes the degradation of the mitochondrial protein PGAM5, increasing DRP1 phosphorylation at S637 and reducing mitoROS generation, thereby suppressing GSDME-mediated pyroptosis and antitumor immune responses.^140^ Various mitochondria-targeting approaches are being developed to induce cancer cell pyroptosis.^141^

#### Necroptosis

Necroptosis represents a regulated form of necrosis that is distinct from apoptosis in terms of morphological features and inflammatory involvement. When apoptosis is inhibited, necroptosis typically initiates through the activation of death receptors, such as the TNF receptor. This activation leads to necrosome complex formation involving receptor-interacting serine/threonine kinase 1 (RIPK1) and RIPK3, resulting in necrotic cell death.^142^ Mixed lineage kinase domain-like pseudokinase (MLKL), the key executor of necroptosis, undergoes RIPK3 phosphorylation and translocates to the plasma membrane, disrupting membrane integrity and releasing cellular contents.^142^ This release exacerbates inflammation, causing tissue injury under pathological conditions. Studies have shown that mitoROS generation promotes RIPK3-mediated necroptosis via the ROS/JNK/Bax pathway.^137^ An imbalance in mitochondrial dynamics plays crucial roles in necroptosis, such as DRP1-mediated mitochondrial fission, which promotes hepatocyte necroptosis.^143^ Additionally, a recent study revealed that NR4A1 depletion inhibits colorectal cancer progression by promoting RIPK3-dependent necroptosis via the RIG-I-like receptor pathway, further highlighting the importance of modulating necroptosis for cancer therapy.^144^

In summary, mitochondrial structural organization, quality control mechanisms, and their regulatory roles in cell death pathways represent fundamental aspects of cellular homeostasis. Dysregulation of these mitochondrial functions has been implicated in various cancers, where altered mitochondrial dynamics and impaired cell death pathways contribute to tumor progression and therapeutic resistance.

## Mitochondrial metabolism and stress adaptation in cancer

**Fig. 3:**
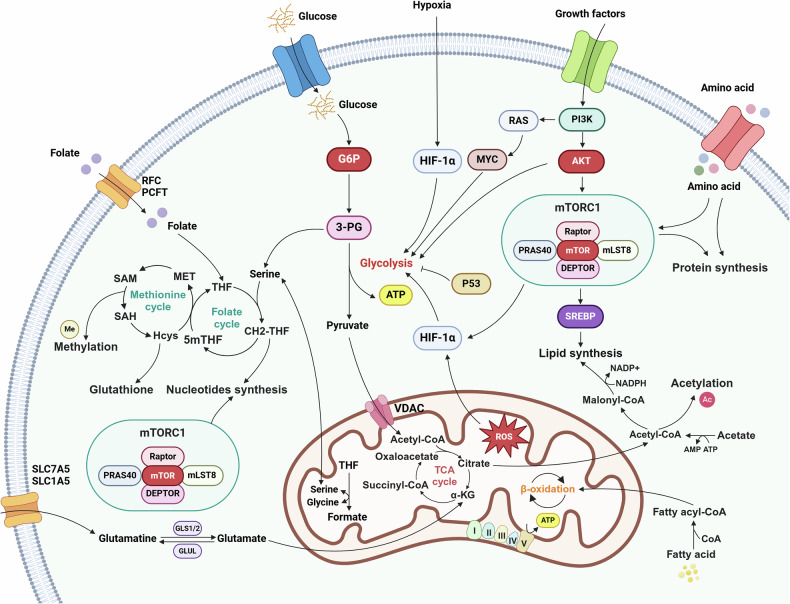
Mitochondrial metabolic and stress adaptations in cancer. Cancer cells develop sophisticated metabolic strategies to sustain proliferation under challenging conditions. These adaptations include increased glucose uptake (Warburg effect), increased glutamine metabolism to replenish energy cycles, the activation of one-carbon metabolism for biosynthesis, and mitochondrial reprogramming to support lipid production and redox balance. By dynamically adjusting metabolic pathways, cancer cells can generate essential metabolic intermediates, maintain bioenergetic efficiency, and survive in nutrient-poor or hypoxic environments that typically inhibit cellular growth and function. 3-PG 3-phosphoglycerate, 5mTHF 5-methyltetrahydrofolate, AMP adenosine monophosphate, ATP adenosine triphosphate, CH2-THF 5,10-methylene tetrahydrofolate, GLUL glutamine synthetase, G6P glucose-6-phosphate, HIF-1α hypoxia-inducible factor 1-alpha, MET methionine, mTORC1 mechanistic target of rapamycin complex 1, mLST8 mammalian lethal with SEC13 protein 8, MYC MYC Proto-Oncogene, NADPH nicotinamide adenine dinucleotide phosphate, OXPHOS oxidative phosphorylation, PI3K phosphatidylinositol 3-kinase, ROS reactive oxygen species, SAH S-adenosyl homocysteine, SAM S-adenosyl methionine, SLC1A5 solute carrier family 1 member 5, SLC7A5 solute carrier family 7 member 5, SREBP sterol regulatory element-binding protein, TCA cycle tricarboxylic acid cycle, THF tetrahydrofolate, VDAC voltage-dependent anion channel, α-KG alpha-ketoglutarate. This figure was created with BioRender (https://biorender.com/)

Malignant transformation is characterized by profound metabolic reprogramming, where cancer cells exhibit remarkable plasticity in their bioenergetic and biosynthetic pathways to sustain rapid proliferation and survival. At the core of this metabolic reorganization lies extensive alterations in mitochondrial function, fundamentally distinguishing cancer cells from normal cells. This reprogramming encompasses comprehensive changes in metabolic enzyme properties, upstream regulatory mechanisms, and downstream metabolite profiles (Fig. 3).

### Warburg effect

The Warburg effect represents a fundamental metabolic hallmark of cancer cells and is characterized by increased glucose uptake and lactate fermentation, even under aerobic conditions. Despite lower ATP production than OXPHOS does, this seemingly paradoxical metabolic phenotype provides advantages for rapidly proliferating malignant cells.

#### Aerobic glycolysis

The molecular control of cancer cell aerobic glycolysis is predominantly governed by a network of oncogenic signaling pathways, with PI3K/AKT and MYC serving as core regulatory factors. These pathways synergistically upregulate glucose transporter and glycolytic enzyme expression, driving cancer cell metabolic reprogramming.^145^ The metabolic network’s core effector molecules focus on three critical rate-limiting enzymes: hexokinase 2 (HK2), phosphofructokinase-1 (PFK-1), and pyruvate kinase M2 (PKM2). These enzymes precisely regulate glycolytic processes through a cascading mechanism. HK2 catalyzes initial glucose phosphorylation, PFK-1 controls irreversible glycolytic steps through allosteric effects, and PKM2 achieves terminal metabolic flux regulation via dynamic tetramer/dimer transitions.^146^ Notably, this cascading regulatory mode is closely associated with dynamic changes in the TME.

Recent research has revealed the intricate multilevel regulatory networks controlling these rate-limiting enzymes in tumors. At the expression level, HK2 tissue-specific expression is induced by microenvironmental factors such as IDO1 (pancreatic cancer) and STING (colorectal cancer), while simultaneously achieving dynamic regulation through the Keap1/Nrf2/Bach1 oxidative stress response axis (hepatocellular carcinoma) and the circCDKN2B-AS1/IMP3 epigenetic regulatory pathway (cervical squamous cell carcinoma).^147^ In terms of epigenetic regulation, the catalytic function of PFK-1 is controlled by USP35-mediated ubiquitination (breast cancer) and dephosphorylation by protein phosphatase 4 (PP4),^148^ whereas the activity state of PKM2 depends on H2S-induced cysteine modification (NSCLC) and CIP2A-mediated oligomerization state transition.^149^ Particularly noteworthy are the characteristics of these metabolic enzymes beyond their classical catalytic functions. HK2 not only acts as a glucose sensor regulating energy homeostasis but also promotes tumor immune evasion through IκBα phosphorylation,^150^ and enhances small cell lung cancer stem cell properties via the USP11-CD133 signaling axis.^151^

These discoveries not only deepen our understanding of aerobic glycolysis regulatory networks but also, more importantly, reveal the multifaceted roles of metabolic enzymes in the regulation of cellular function. These findings provide novel insights for developing innovative metabolism-targeted therapeutic strategies.

#### Lactate production

The biological functions of lactate in the TME have transcended traditional metabolic understanding, revealing its characteristics as a multifunctional signaling molecule. Research has demonstrated that cancer cells generate substantial amounts of lactate through lactate dehydrogenase (LDH)-mediated glycolysis.^152^ These metabolic products are exported via monocarboxylate transporter systems, creating an acidic microenvironment that directly activates pro-invasive signaling pathways and induces matrix metalloproteinase expression, thereby increasing metastatic potential. Notably, lactate has a dual immunomodulatory effect. Extracellular lactate weakens antitumor immune responses by suppressing the glycolytic capacity of CD8^+^ T cells and interfering with IFN-γ signal transduction.^153^ Conversely, specific lactate concentrations can reshape myeloid cell metabolic programs to promote immunosuppressive microenvironments.^154^ More importantly, lactate regulates tumor biological behaviors through nonmetabolic pathways. In cervical cancer, it enhances the formation of cell adhesion pseudopodia via the β-catenin/fascin signaling axis, establishing a direct link between metabolic reprogramming and cytoskeletal dynamics.^155^

Lactate dehydrogenase A (LDHA), a core executor of the Warburg effect, is involved in a multilayered functional regulatory network. LDHA maintains the NAD^+^/NADH redox balance to ensure glycolytic flux,^156^ and lactate accumulation significantly correlates with adverse outcomes in multiple malignancies. Several studies have revealed the noncanonical functions of LDHA. In breast cancer, it reshapes cell motility through RAC1 GTPase activation,^157^ whereas in pancreatic cancer, it induces neutrophil polarization to form immunosuppressive ecosystems.^158^ METTL3 precisely controls LDHA expression in colorectal cancer through dual mechanisms: stabilizing hypoxia inducible factor 1 subunit alpha (HIF-1α) mRNA to increase transcription and promote translation via the m^6^A-YTHDF1 axis.^159^ In hepatocellular carcinoma, the circFOXK2-FOXK2-142aa pathway regulates LDHA activity through phosphorylation, triggering cascading mitochondrial division and metabolic reprogramming.^160^ These findings suggest that LDHA functions not only as a metabolic hub but also as a critical node connecting epigenetic and organelle dynamic regulation.

The discovery of protein lactylation has inaugurated a new dimension of metabolic epigenetic control. Research has confirmed that lactate can function as an endogenous lactyl donor. NSUN2 lactylation enhances GSH synthesis to resist ferroptosis,^161^ whereas PIK3C3/VPS34 lactylation influences tumor progression through autophagy activation.^162^ In epigenetic reprogramming, H3K18 lactylation in colorectal cancer induces bevacizumab resistance through RUBCNL,^163^ whereas CBX3-mediated histone lactylation in glioblastoma reshapes immune checkpoint expression.^164^ Notably, the glycolysis-lactylation feedback loop discovered in pancreatic ductal adenocarcinoma and global lactylation mediated by alanyl-TRNA synthetase 1/2 (AARS1/2), which regulate the cGAS-p53 signaling axis, reveal an intricate metabolic-epigenetic cross-regulatory network.^165^ These findings not only expand the functional boundaries of lactate but also suggest that targeting lactate metabolism requires consideration of its dual attributes as both a metabolite and a signaling molecule.

### Mitochondrial respiration in cancer: integration of OXPHOS and TCA cycle dynamics

#### Oxidative phosphorylation

The dynamic equilibrium between OXPHOS and glycolysis constitutes the core characteristic of metabolic plasticity. Research indicates that despite aerobic glycolysis being the primary energy source for most tumors, OXPHOS plays a critical role in tumor progression under specific conditions.^166^ From an energy perspective, glycolysis generates 2 ATP molecules per glucose molecule, whereas OXPHOS can produce up to 36 ATP molecules.^167^ Researchers have noted that cancer cells can dynamically oscillate between glycolysis and OXPHOS on the basis of microenvironmental conditions and cellular demands, emphasizing the concept of metabolic plasticity.^168^

This flexibility stems from the dynamic regulation of the glycolysis/OXPHOS balance in cancer cells through molecular switches such as HIF-1α. HIF-1α not only upregulates glycolytic enzyme expression but also reduces OXPHOS activity by inhibiting mitochondrial biogenesis.^169^ PKM2 coordinates mitochondrial fusion and OXPHOS progression through interaction with c-Myc,^170^ and its nuclear localization promotes metabolic reprogramming by suppressing H2Bub1 modification.^171^ Notably, the differential subcellular localization of MYG1 reveals a novel spatial regulatory mechanism. Nuclear MYG1 enhances glycolysis by stabilizing PKM2, whereas mitochondrial MYG1 directly suppresses OXPHOS, with this nuclear-mitochondrial coordination emerging as a key mechanism in metabolic remodeling in colorectal cancer.^172^ This multilayered regulatory network enables cancer cells to rapidly switch metabolic modes under stress.

Tumor metabolic heterogeneity manifests significant variations across tissue types and cellular subpopulations. Studies have demonstrated that NSCLC simultaneously enhances glycolysis and glucose oxidation.^173^ High-OXPHOS melanoma subtypes exhibit synergistic activation of dual metabolic pathways.^174^ Leukemia stem cells (LSCs) are OXPHOS dependent, suggesting potential therapeutic vulnerability.^175^ This diversity provides evolutionary selective advantages. Malignancies such as pancreatic ductal adenocarcinoma maintain dual glycolysis/OXPHOS activity to adapt to nutritional fluctuations, whereas acute myeloid leukemia (AML) cells utilize OXPHOS to maintain stemness. Metabolic heterogeneity extends beyond cancer cells to the entire TME. Cancer-associated fibroblasts (CAFs) enhance glycolysis reprogramming through circABCC4-mediated PKM2 nuclear translocation to promote oxaliplatin resistance in pancreatic cancer.^176^ Th17 cell OXPHOS activation can improve antitumor efficacy.^177^ Stromal stiffness enhances tumor-infiltrating Treg cells (TI-Tregs) OXPHOS via the YAP-Lars2 axis, revealing crosstalk between mechanical signaling and metabolic reprogramming.^178^

Metabolic symbiosis also frequently occurs in the TME. Circulating tumor cells (CTCs) acquire metastatic advantages through OXPHOS activation,^179^ whereas lactate secreted by glycolytic cells can nourish adjacent OXPHOS-dependent cells through a “reverse Warburg effect”.^180^ This metabolic interaction demonstrates bidirectional immunomodulation. The shift of macrophages toward glycolysis can enhance antitumor immunity,^181^ while tumor-derived lactate simultaneously reshapes myeloid metabolism to promote immunosuppression. Recent intervention strategies targeting metabolic symbiosis nodes, specifically a low-leucine diet combined with YAP inhibitors, can effectively disrupt the TI-Treg OXPHOS-immunosuppression axis by inhibiting the YAP-Lars2-leucine pathway, suggesting novel approaches to overcome tumor microenvironment-mediated immunosuppression and therapeutic resistance.^178^

#### TCA cycle

The central role of the TCA cycle in tumor metabolism stems from its multidimensional regulatory characteristics, with its reconstruction involving not only classical enzyme activity modulation but also metabolic flux redirection and the establishment of novel metabolite networks. Cancer cells ingeniously balance energy generation and biosynthetic demands by operating the cycle discontinuously, complementing glutamine metabolism, and initiating α-ketoglutarate reverse carboxylation under hypoxic conditions.^182^ This metabolic plasticity is established through mitochondrial redox regulatory pivots. The TCA cycle serves as the primary source of electron transfer chain-reducing equivalents and generates NADPH through IDH reactions to maintain antioxidant defense.^183^ Metabolites such as α-ketoglutarate directly regulate the NAD^+^/NADH ratio, influencing oxidation-reduction-sensitive signaling pathways.^184^ Notably, the integration of the TCA cycle with one-carbon metabolism and GSH systems results in a cross-compartmental redox buffering network, potentially representing a critical evolutionary advantage in tumor metabolic stress resistance.^185^

Cancer cells remodel the TCA cycle through multilayered regulatory networks. YAP enhances TCA activity by increasing Lars2 expression, whereas ARID1A deletion forces metabolic flux toward the TCA cycle by suppressing PKM.^186^ IscU2 stabilizes Fe-S clusters to activate α-ketoglutarate dehydrogenase,^187^ and MPST deletion directly inhibits TCA progression.^188^ Furthermore, P4HA1 accumulation in mitochondria can disrupt the α-ketoglutarate/succinate metabolic balance, with TCA cycle intermediates demonstrating dual metabolic-epigenetic functions.^189^ α-Ketoglutarate acts as an epigenetic regulator affecting demethylase activity, whereas citrate reshapes chromatin structure through ACLY-mediated histone acetylation.^190^ These findings suggest that the TCA cycle has become a critical interface connecting metabolic reprogramming and epigenetic regulation.

Mutations in key TCA cycle enzymes constitute metabolic drivers of tumorigenesis. IDH mutations have been the most extensively studied, with a high prevalence in gliomas and AML, providing a classic example of metabolite-epigenetic regulation.^191^ Research has revealed that IDH mutations induce genome-wide epigenetic changes by generating D-2-hydroxyglutarate (D-2-HG), leading to DNA and histone methylation phenotype alterations.^192^ IDH mutations can also remodel the TME, affecting the frequency of tumor-infiltrating lymphocytes and modulating innate immune responses through ATRX and IDH1 mutation interactions.^193^ Moreover, these mutations impact tumor progression by influencing ribosomal biology and heterochromatin-associated replication stress.^194^ Equally noteworthy are SDH and FH mutations. SDH subunit mutations cause succinate accumulation, which is closely associated with paragangliomas and pheochromocytomas.^195^ SDH deficiency impacts mitochondrial function and oxidative stress, drives tumorigenesis through chromatin topological changes, and is related to homologous recombination DNA repair defects.^196^ FH germline mutations lead to hereditary leiomyomatosis and renal cell cancer (HLRCC).^197^ FH deletion causes fumarate accumulation, which induces cysteine succinylation modifications affecting protein function and triggers innate immune responses by inducing mtDNA release.^198^ Recent therapeutic strategy breakthroughs include the discovery of synthetic lethal targets such as CHD6 in FH-deficient tumors,^199^ and the role of HIRA deletion in promoting FH-mutated cell transformation,^200^ suggesting new directions for targeting metabolically mutated tumors.

### Substrate utilization in cancer metabolism

Cancer cells exhibit profound changes in substrate utilization patterns, distinguishing them from normal cells. This metabolic reprogramming enables their survival and proliferation in nutrient-deficient, hypoxic, and stress-laden microenvironments.

#### Glutamine metabolism

Glutamine metabolism represents a critical aspect of cancer cell adaptation and survival. As the most abundant amino acid in circulation, glutamine serves not only as a nitrogen source but also as a crucial metabolic substrate that supports increased proliferative needs and survival mechanisms through glutaminolysis. Through this process, glutamine is converted to glutamate, which is then deaminated by glutamate dehydrogenase (GDH) or transaminases, forming α-KG, which enables continuous TCA cycle function even under glucose-limited conditions.^201^

In energy metabolism, glutamine can function as an alternative energy source when the glucose supply is restricted, promoting its breakdown through the upregulation of mitochondrial cytochrome C oxidase II (MT-CO2) to maintain cancer cell survival.^202^ Within the metabolic regulatory network, glutamine utilization is subject to multiple controls. The m^6^A reader IGF2BP2 regulates glutamine metabolism by modulating key genes, including MYC, GPT2, and SLC1A5.^203^ SLC25A15 deletion can increase glutamine uptake by upregulating SLC1A5.^204^ In KRAS-mutated tumors, metabolic networks are reshaped by promoting glutamine absorption.^205^

With respect to metabolic enzyme activity regulation, filamentous formation of glutaminases (GAC and GLS2) plays a crucial role in their catalytic activity.^206^ Moreover, glutamine metabolism impacts lipid synthesis through ammonia release. During glutamine breakdown, ammonia can activate sterol regulatory element binding transcription factor 1 (SREBP1), thereby promoting lipid synthesis and tumor growth.^207^ In pancreatic cancer cells, glutamine supports polyamine synthesis through the ornithine synthesis pathway.^208^ In metabolic adaptability, the m^6^A RNA methyltransferase METTL16 participates in glutamine biosynthesis by regulating GLUL expression,^209^ whereas interferon-related developmental regulator 1 (IFRD1) can modulate cancer cell survival under glutamine deprivation by suppressing histone H1.0 nucleophagy.^210^ This multilayered regulatory mechanism ensures that cancer cells can flexibly adjust glutamine metabolism according to nutritional status to support growth and proliferation.

Glutamine metabolism reprogramming also involves cooperative interactions across multiple signaling pathways. Studies have demonstrated that glutamine metabolism can regulate tumor growth and survival by influencing AMPK signaling, the PI3K/AKT pathway, and the STAT1 signaling pathway.^211^ In particular, under stress conditions such as glucose deficiency, cancer cells maintain survival by increasing glutamine metabolism-related enzyme and transporter expression.^212^ Furthermore, glutamine metabolism is closely connected with cell cycle regulation. The cell cycle-specific transcription factor E2F can directly regulate glutamine metabolism-related gene expression,^213^ while glutamine metabolites can, in turn, affect cell cycle protein stability. This bidirectional regulatory mechanism ensures precise matching between cell proliferation and metabolic demands. Additionally, glutamine can also modulate epigenetic modifications. Glutamine metabolism can influence histone demethylase activity through α-KG, which participates in epigenetic regulation.^200^ The coupling of glutamine metabolism with one-carbon metabolism can affect DNA and histone methylations.^214^ These findings significantly advance our understanding of the role of glutamine in tumor metabolic reprogramming and offer new perspectives on tumor cell metabolic plasticity.

#### Lipid metabolism

Cancer cells exhibit remarkable adaptability in lipid metabolism, fundamentally altering their physiological functions to support rapid proliferation, enhanced migration, and antiapoptotic mechanisms. These metabolic modifications encompass multiple aspects of lipid synthesis, storage, oxidation, and signaling pathways, collectively contributing to the invasive phenotypic characteristics of malignant cells.

A hallmark of cancer cells is the accumulation of cytoplasmic lipid droplets, which provide diverse critical functions, including energy storage under nutrient-restricted conditions, prevention of lipotoxicity, and regulation of cellular stress responses.^215^ Studies have established correlations between lipid droplet abundance and enhanced metastatic potential across various cancer types.^216^ Moreover, these lipid metabolic adaptations play crucial roles in maintaining cancer stem cells (CSCs), where the inhibition of fatty acid oxidation weakens their self-renewal capacity and increases their chemotherapeutic sensitivity.^217^ Deletion of carnitine palmitoyltransferase 1a (CPT1A), a key enzyme in fatty acid oxidation, leads to increased mitochondrial respiratory chain complex components and activity, increased ATP production, and mitoROS accumulation, causing hematopoietic stem cell (HSC) dysfunction.^218^ Cancer cells also demonstrate modified lipid signaling pathways involving bioactive lipids such as prostaglandins, sphingolipids, and eicosanoids, which regulate inflammatory responses, tumor progression, metastatic potential, and cell survival mechanisms, collectively contributing to the complex landscape of cancer metabolism.^219^

The demand for rapid cell proliferation drives extensive membrane biogenesis through enhanced de novo lipogenesis, wherein cancer cells redirect glucose and other metabolic intermediates toward fatty acid and phospholipid synthesis. Notably, quantitative analyses indicate that a significant portion of triglycerides in cancer cells originate from de novo synthesis rather than from external sources.^220^ Moreover, fatty acid oxidation has emerged as a critical energy source, particularly under metabolic stress conditions. Cancer cells utilize fatty acid oxidation to generate ATP and NADPH, which are crucial for maintaining redox balance and supporting synthetic metabolic processes.^221^

A primary characteristic of cancer-associated lipid metabolism is the significant upregulation of fatty acid synthesis, primarily through the increased activity of key enzymes, including fatty acid synthase (FASN) and acetyl-CoA carboxylase (ACC).^222^ FASN overexpression, which has been documented across various cancer types, is significantly correlated with disease progression and adverse clinical outcomes.^223^ Within the metabolic regulatory network, FASN modulates protein stability through interactions with ZDHHC21 and palmitoylation modifications,^224^ and regulates cell growth and proliferation via the mTOR signaling pathway. In metabolic reprogramming, abnormal FASN activation not only promotes de novo fatty acid synthesis but also regulates cancer cell biological properties by influencing membrane lipid composition.^225^ In particular, FASN activation is critical for maintaining cancer cell survival in KRAS-mutated tumors.^226^ Furthermore, FASN participates in cellular energy metabolism by regulating mitochondrial function. The expression level of FASN impacts mitochondrial dynamic equilibrium and OXPHOS and modulates ferroptosis sensitivity by influencing lipid peroxidation.^227^ Similarly, ACC1 controls lipid droplet-peroxisome axis in endocrine-resistant ER^+^ breast cancer, establishing it as a therapeutic vulnerability.^228^ In prostate cancer, CircPCNXL2 promotes tumor progression by interacting with ACC1 to affect fatty acid metabolism and enhance cell growth.^229^ Additionally, ACC obstructs CD8^+^ T cell lipid utilization in the tumor microenvironment.^230^ These findings substantially illuminate our understanding of cancer cell lipid metabolism and offer novel perspectives for targeted therapeutic interventions.

#### One-carbon metabolism

One-carbon metabolism constitutes a critical metabolic axis that supports cancer cell proliferation and survival by coordinating numerous processes involved in cellular biosynthesis, redox regulation, and epigenetic control. This multifaceted pathway, encompassing the folate and methionine cycles, enables cancer cells to synthesize and utilize single-carbon moieties for fundamental biological functions, including nucleotide biosynthesis, DNA methylation, and redox balance.^231^ Compared with normal cells, malignant cells demonstrate significantly elevated one-carbon metabolic activity, reflecting their increased demand for precursor molecules and regulatory adaptability.

Within this network, the folate cycle is an indispensable component for generating and consuming single-carbon units, particularly for purine and thymidine biosynthesis. Key enzymes include serine hydroxymethyltransferase (SHMT) and methylenetetrahydrofolate dehydrogenase (MTHFD1). SHMT catalyzes serine and glycine interconversion to provide essential methyl groups, whereas MTHFD1 produces critical folate intermediates.^232^ Abnormalities in these enzymatic steps can disrupt methylation homeostasis, linking metabolic reprogramming to dynamic cancer epigenomic changes. Recent studies have indicated that SHMT promotes cancer cell survival in KRAS and liver kinase B1 (LKB1) double-mutated NSCLC by increasing serine-glycine-one-carbon (SGOC) metabolism and antioxidant defense.^233^ MTHFD1 maintains the nucleotide balance in cancer cells by regulating folate metabolism and pyrimidine synthesis. Its inhibition leads to 10-formyltetrahydrofolate (10-CHO-THF) accumulation and thymidine synthesis impairment, affecting cancer cell proliferation and survival.^232^

Parallel to the folate cycle, the methionine cycle produces S-adenosylmethionine (SAM), a critical methyl donor for DNA, RNA, and histone methylation. SAM synthesis is catalyzed by methionine adenosyltransferase (MAT), with downstream byproducts such as S-adenosylhomocysteine (SAH) providing feedback inhibition to ensure strict methylation dynamics regulation.^234^ Consequently, cycle disruptions can trigger hyper- and hypomethylation events with profound implications for tumorigenesis. The variations in SAM levels in cancer cells affect histone H3K79 dimethylations and tumor progression through LINC00662-mediated epigenetic regulation.^235^ In hepatocellular carcinoma, methionine cycle dysregulation alters SAM levels, subsequently impacting genome-wide methylation. Moreover, changes in SAM levels can regulate tumor growth by influencing PRMT5-dependent mRNA splicing and DNA damage responses,^236^ whereas SAM/SAH ratio modifications affect cancer cell methylation states.

In addition to nucleotide synthesis and epigenetic regulation, one-carbon metabolism promotes redox homeostasis by generating NADPH and GSH.^237^ In proliferating cancer cells, one-carbon metabolism represents the second-largest intracellular NADPH source after the pentose phosphate pathway, with tetrahydrofolate oxidation generating substantial amounts of NADPH.^237^ The NADPH produced through one-carbon metabolism is crucial for maintaining redox balance and supporting biosynthetic processes.^238^ In conclusion, one-carbon metabolism has emerged as a key nexus that integrates metabolic, epigenetic, and regulatory circuits. The synergistic interactions of this metabolic network provide cancer cells with metabolic plasticity and survival advantages.

### Stress response programs in cancer metabolism

#### Hypoxia

Cancer cells encounter a series of environmental stressors within the TME, with hypoxia posing a significant threat because of its profound impact on cellular metabolism. The HIF pathway mediates primary adaptive responses, coordinating a transcriptional program that promotes survival under hypoxic conditions. Upon low-oxygen-induced stabilization, HIF-1α translocates to the nucleus, where it upregulates genes involved in cellular metabolism, angiogenesis, and invasion, providing cancer cells with mechanisms to cope with a reduced oxygen supply.^239^

Research has demonstrated that HIF stability is precisely regulated through multiple mechanisms. Oxygen-dependent proline and asparagine residue hydroxylation modifications can modulate HIF protein stability and transcriptional activity.^240^ The ubiquitin ligase VHL regulates HIF stability by recognizing hydroxylated HIF-α and mediating its ubiquitin-dependent degradation. Furthermore, cancer cells employ additional molecular mechanisms to regulate HIF stability. Hydrogen sulfide modulates HIF-1α stability through persulfidation of PHD2,^241^ whereas peptidyl arginine deiminase 4 (PADI4) promotes HIF-1α stabilization through citrullination modification.^242^ Notably, HIF stabilization can impact other cancer-associated pathways through feedback regulation. In gliomas, HIF can promote tumor progression by regulating c-Myc transcriptional activity.^243^ Mutations in TCA cycle enzymes such as SDH can lead to metabolite accumulation (such as succinate), inhibiting prolyl hydroxylases and affecting HIF-1α stability.^244^ These mechanisms collectively promote a shift in cellular metabolism toward glycolysis, reducing the dependence on OXPHOS and conferring survival advantages under hypoxic conditions.

Several mitochondrion-localized or mitochondria-associated regulatory proteins also influence HIF stability. The mitochondrial deacetylase SIRT3 regulates ROS levels, thereby affecting HIF-1α stability and overall metabolic reprogramming.^245^ Similarly, SIRT4 contributes through its impact on ROS-mediated HIF-1α stabilization.^246^ Additionally, mitochondrial UQCC3 maintains HIF-1α stability by modulating ROS production, thereby influencing cancer cell metabolic adaptation.^247^ These complex interactions underscore the critical role of mitochondrial function in regulating HIF-mediated responses.

#### Nutrient stress response

Cancer cells employ complex stress response mechanisms to combat nutrient deprivation, engaging multiple adaptive pathways to ensure survival under challenging metabolic conditions. These responses include coordinated modifications of protein synthesis, energy metabolism, and lipid utilization, collectively enabling cellular adaptation to nutritional stress.

In response to amino acid deficiency, cancer cells activate the integrated stress response (ISR), a conserved adaptive pathway centered on eIF2α phosphorylation and subsequent ATF4 activation.^248^ This stress response mechanism is closely associated with mitochondrial dysfunction and oxidative stress. The response coordinates a comprehensive transcriptional program regulating protein synthesis and cellular metabolism. Mitochondrial protein misfolding can trigger the mitochondrial unfolded protein response, which is closely linked to ISR pathway activation. Moreover, the ISR can influence mitochondrial function by regulating amino acid transporter expression.^249^ Amino acid sensors, particularly GCN2 and mTORC1, play critical roles in this adaptation, serving as molecular switches that modulate protein synthesis rates and metabolic pathways on the basis of nutrient availability.^250^

Glucose starvation triggers distinct adaptive mechanisms characterized by autophagy induction and alternative degradative pathway activation.^251^ Despite limited glucose availability, this metabolic reprogramming ensures the maintenance of cellular energy. Central to this response is the activation of AMPK, the primary regulator of cellular energy homeostasis.^251^ AMPK activation initiates a series of metabolic adaptations, including the inhibition of energy-intensive biosynthetic processes,^252^ and the suppression of lipid peroxidation-related ferroptosis,^253^ thereby maintaining cell viability under glucose-limited conditions. Mitochondria play a crucial role in this process, reshaping metabolic networks to maintain energy balance. For example, PKM2 mitochondrial translocation can promote cell survival during glucose starvation.^254^ In colorectal cancer, the long noncoding RNA GLCC1 is significantly upregulated during glucose starvation, supporting cell survival by stabilizing c-Myc.^255^ Specific molecules, such as mesencephalic astrocyte-derived neurotrophic factor (MANF), can promote breast cancer cell survival under glucose deprivation conditions by regulating mitochondrial autophagy.^256^

Under lipid deficiency, cancer cells activate lipophagy, a specialized form of autophagy that targets cellular lipid stores.^257^ This process mobilizes stored lipids to support cellular energy requirements and membrane biogenesis. Lipid metabolic pathways simultaneously undergo modifications, including changes in fatty acid synthesis, oxidation, and lipid-mediated signaling.^257^ During nutrient deprivation, SREBP2 regulates cholesterol biosynthesis and uptake in glioblastoma stem cells.^258^ McLelland et al. reported that the acyltransferase TMEM68 can obtain fatty acids from cell membrane phospholipids, synthesize triglycerides under specific conditions, and maintain mitochondrial function during external lipid scarcity.^259^ Moreover, mitochondrial lipid metabolic remodeling is crucial for cancer cell survival, maintaining energy balance by regulating fatty acid oxidation and mitochondrial function.^260^

These adaptations reflect the remarkable metabolic flexibility of cancer cells, enabling them to maintain fundamental cellular functions under nutrient-deprived conditions.

#### Redox homeostasis

Cancer cells exhibit complex mechanisms for maintaining redox homeostasis and balancing ROS production to support survival and proliferation. The intricate relationships among ROS generation, antioxidant defense, and cell signaling pathways represent critical aspects of cancer cell metabolism and stress adaptation.

Mitochondrial respiration is the primary source of ROS in cancer cells, producing superoxide and hydrogen peroxide as metabolic byproducts. These ROS molecules play a dual role, influencing key cellular processes, including gene expression, cell cycle regulation, and DNA repair mechanisms.^261^ While moderate ROS levels can promote oncogenic signaling and survival pathways, excessive ROS accumulation may trigger oxidative damage and apoptotic responses, necessitating complex regulatory mechanisms. Mechanistically, NADPH is a critical coenzyme in maintaining redox homeostasis,^262^ whereas the Nrf2 transcription factor plays a core role in regulating antioxidant gene expression. Abnormal redox homeostasis regulation is closely associated with malignant progression across tumor types. In colorectal cancer, the USP11/Nrf2 positive feedback loop promotes tumor progression by suppressing mitochondrial apoptosis.^263^ In melanoma, mitochondrial calcium regulation influences redox homeostasis and phenotypic transition.^264^ Additionally, telomerase reverse transcriptase (TERT) performs atypical functions in maintaining redox homeostasis, protecting cancer cells from apoptosis.^265^ Consequently, redox homeostasis regulation not only affects cancer cell metabolism and survival but also may provide novel therapeutic targets and strategies.

In summary, mitochondria have emerged as master regulators of cancer metabolic reprogramming, transcending their traditional role as energy producers to become dynamic signaling platforms. By orchestrating redox homeostasis, metabolic plasticity, and stress responses, these versatile organelles precisely coordinate cancer cell survival strategies, offering intricate molecular mechanisms that underpin tumor progression and therapeutic resistance.

## Mitochondrial signaling networks in cancer

### Metabolic sensors

**Fig. 4:**
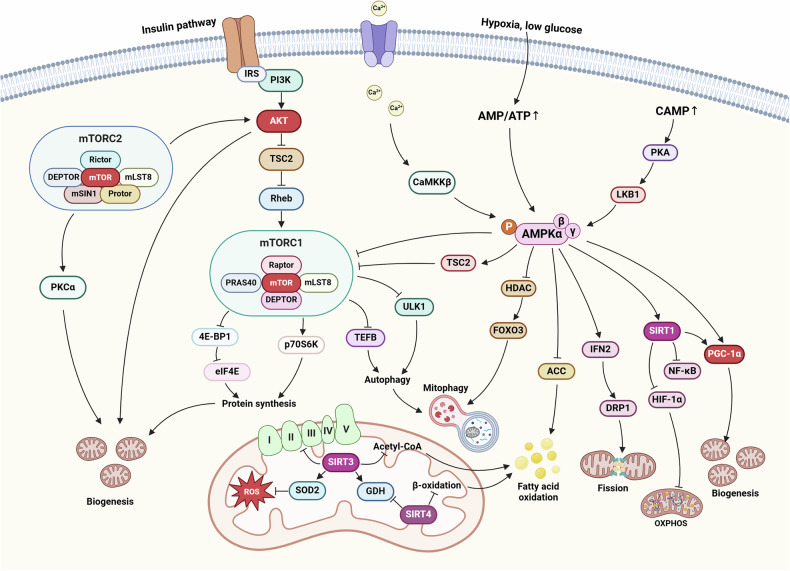
Signaling networks regulating mitochondrial function and adaptation in cancer cells. Cancer cell survival depends on complex signaling networks that dynamically regulate mitochondrial function. The insulin pathway activates PI3K and AKT, stimulating mTORC1 to drive cellular growth, whereas AMPK serves as a critical metabolic sensor under stress conditions. When activated, AMPK modulates mitochondrial dynamics, autophagy, and energy metabolism by interacting with key proteins such as ULK1, SIRT1, and PGC-1α. Mitochondrial proteins such as SIRT3 further contribute to cellular adaptation by managing oxidative stress and maintaining metabolic balance. These interconnected pathways enable cancer cells to survive and adapt to challenging environments, suggesting potential targets for therapeutic intervention. 4E-BP1 eIF4E-binding protein 1, ACC acetyl-CoA Carboxylase, AKT protein kinase B, AMP adenosine monophosphate, AMPK AMP-activated protein kinase, ATP adenosine triphosphate, CaMKKβ calcium/calmodulin-dependent protein kinase kinase β, cAMP cyclic adenosine monophosphate, DEPTOR DEP domain-containing mTOR-interacting protein, DRP1 dynamin-related protein 1, eIF4E eukaryotic translation initiation factor 4E, FOXO3 forkhead box O3, GDH glutamate dehydrogenase, HDAC histone deacetylase, HIF-1α hypoxia-inducible factor 1-alpha, IFN2 interferon 2, IRS insulin receptor substrate, LKB1 liver kinase B1, mLST8 mammalian lethal with SEC13 protein 8, mTOR mammalian target of rapamycin, mTORC1/2 mTOR complex 1/2, mSIN1 mammalian stress-activated protein kinase interacting protein 1, NF-κB nuclear factor kappa B, OXPHOS oxidative phosphorylation, p70S6K 70 kDa ribosomal protein S6 kinase, PGC-1α peroxisome proliferator-activated receptor gamma coactivator 1-alpha, PI3K phosphatidylinositol 3-kinase, PKA protein kinase A, PKCα protein kinase C alpha, Rheb Ras homolog enriched in brain, ROS reactive oxygen species, SIRT1/3/4 Sirtuin 1/3/4, SOD2 superoxide dismutase 2, TEFB transcription elongation factor B, TSC2 tuberous sclerosis complex 2, ULK1 UNC-51-like kinase 1. This figure was created with BioRender (https://biorender.com/)

Mitochondrial signaling networks integrate metabolic states with cancer progression through key metabolic sensors such as AMPK, mTOR, and Sirtuins, coordinating cellular energy metabolism during malignant transformation (Fig. 4).

#### AMPK signaling

AMPK is a highly conserved heterotrimeric complex that serves as the primary cellular energy sensor. AMPK activation occurs through a two-step mechanism. Under energy stress conditions (such as glucose deprivation or hypoxia), the binding of AMP or ADP to the γ regulatory subunit induces a conformational change in the AMPK complex, increasing its sensitivity to upstream kinases. The second step involves phosphorylation of the α catalytic subunit at the activation loop site (Thr172) by upstream kinases, primarily LKB1 and CaMKKβ.^266^ Phosphoproteomic analyses have identified over 50 direct AMPK substrates, including the key metabolic enzymes acetyl-CoA carboxylase (ACC) and HMG-CoA reductase. Notably, AMPK-mediated phosphorylation of PGC-1α coordinates mitochondrial biogenesis and function by upregulating the transcriptional programs of genes involved in OXPHOS and mtDNA replication.^97^ Inositol can directly bind to AMPKγ to competitively inhibit its activity, regulating mitochondrial dynamics.^267^

AMPK plays a dual role in mitochondrial function. On the one hand, it acts as a tumor suppressor by inducing autophagy, inhibiting energy metabolism, and modulating oxidative stress. Conversely, in specific TMEs, AMPK may promote cancer cell survival and metastasis. In AML, the AMPK-PERK axis suppresses oxidative metabolism and facilitates the initiation of mitochondrial apoptosis.^268^ Under energy stress, the AMPK/FoxO3a signaling pathway in cancer cells can modulate mitochondrial activity and alter iron death responses.^269^ AMPK phosphorylation of inverted formin 2 (INF2) at Ser1077 increases its endoplasmic reticulum (ER) localization and enhances the recruitment of mitochondrial DRP1, promoting endometrial cancer cell growth.^270^ Under hypoxic conditions, the prolyl hydroxylase EglN1 interacts with AMPKα and accumulates on mitochondria, forming a Ca^2+^/calmodulin-dependent protein kinase 2 (CaMKK2)-EglN1-AMPKα complex to activate AMPKα phosphorylation, ensuring metabolic homeostasis and breast tumor growth.^271^ MitoROS serve as physiological AMPK activators, with AMPK activation triggering a PGC-1α-dependent antioxidant response that limits mitoROS production.^272^ Drake et al. further revealed the mechanism of the mitochondrion-localized AMPK (mitoAMPK) response to local energy dynamics.^273^ These findings underscore that the interaction between AMPK and mitochondria in tumor signal transduction is a dynamic, complex process, providing crucial insights into the mechanisms of cancer initiation and progression.

#### mTOR complexes

mTOR controls cellular metabolism by integrating signals from nutrients, growth factors, and other environmental cues. The two mTOR complexes, mTORC1 and mTORC2, exhibit differential sensitivity to rapamycin and play pivotal roles in regulating the cellular metabolic equilibrium through distinct yet interconnected signaling pathways.

mTORC1, a complex comprising mTOR, Raptor, and mLST8, is sensitive to rapamycin and plays a central role in regulating cellular metabolism. mTORC1 precisely modulates mitochondrial function through multiple regulatory mechanisms. It directly influences mitochondrial oxidative metabolism and biogenesis, regulates mitochondrial gene expression via the PGC-1α and YY1 transcription factors, modulates ETC protein expression, and controls the mitochondrial membrane potential and oxygen consumption.^274^ Notably, mTORC1 hyperactivation suppresses TCA cycle while promoting glycolysis and glutaminolysis.^275^ Rheb can activate pyruvate dehydrogenase (PDH) through mTORC1-independent pathways, modulating the TCA cycle and ATP production.^276^ During mitochondrial stress responses, v-ATPase-mediated lysosomal activation of mTORC1 directly phosphorylates transcription factors, regulating mitochondrial stress responses.^277^ mTORC1 further influences inflammatory cell death and mitochondrial function by modulating ROS generation, revealing its complex role in regulating mitochondrial function.

mTORC2, which is composed of mTOR, Rictor, SIN1, and mLST8, is insensitive to rapamycin, with a more complex and less understood mitochondrial metabolic regulatory mechanism. Research indicates that mTORC2 primarily regulates mitochondrial metabolism through Akt, employing multifaceted mechanisms such as modulating calcium flux and membrane potential at mitochondria-associated endoplasmic reticulum membranes (MAM),^278^ and phosphorylating mitochondria-resident proteins such as ATP synthase subunits. These diverse regulatory strategies underscore the intricate role of mTORC2 in mitochondrial metabolic homeostasis and cellular energetics. Interestingly, the regulatory effects of mTORC2 on mitochondrial metabolism are bidirectional. Some studies suggest that it may impair mitochondrial homeostasis,^279^ whereas others indicate that it can promote mitochondrial function.^280^ This complexity is likely related to the subcellular localization, physiological state, and cell type of mTORC2. In muscle cells, mTORC2 influences muscle fiber types by regulating mitochondrial dynamics and altering mitochondrial fission and accumulation, thereby affecting cellular metabolism.^280^ In macrophages, mTORC2 modulates mitochondrial function by regulating Elp3-mediated protein translation and mitochondrial ribosomal large subunit protein expression.^281^

mTOR complexes play pivotal roles in cancer metabolism through intricate mitochondrial regulatory mechanisms. In chronic myeloid leukemia (CML), mTORC1 senses purine deficiency when mitochondrial folate metabolism is inhibited, triggering differentiation of cancer stem cells and reducing tumor growth.^282^ In AML, mTOR influences cellular metabolism and survival by regulating autophagy.^283^ Similarly, mTORC2 mediates RAS-induced ROS signaling that drives mitochondrial fission, mitophagy and autophagic cell death in KRAS-mutant colorectal cancer.^284^

These studies reveal that mTORC1 and mTORC2 are critical nodes in the regulation of mitochondrial function and play complex and precise regulatory roles during cancer progression.

#### Sirtuins

Sirtuins, an NAD^+^-dependent histone deacetylase family, exhibit remarkable complexity and diversity in molecular regulatory networks related to cellular metabolism and cancer progression.^285^ Despite sharing a conserved catalytic core structure, significant differences in the N-terminal and C-terminal domains reveal nuanced mechanisms of regulatory specialization.^286^ As critical metabolic sensors, Sirtuins demonstrate multilayered regulatory strategies by modulating mitochondrial protein acetylation levels, with their metabolic sensing function particularly noteworthy in energy balance and stress responses.

SIRT1, the most extensively studied sirtuin, has multidimensional regulatory functions in cancer biology. As a key metabolic sensor, its molecular mechanisms encompass mitochondrial function regulation, cellular stress responses, and epigenetic control. In various cancer types, SIRT1 has significant functional diversity. In oral cancer, SIRT1 promotes apoptosis by inhibiting mitochondrial fusion.^287^ In diffuse large B-cell lymphoma, SIRT1 forms a complex feedback loop with HSP90α, precisely regulating chromosome segregation integrity.^288^ Critically, SIRT1 has multiple functions in tumor metabolism and stress regulation. By modulating mitochondrial protein deacetylation to control cellular energy metabolism, it influences drug sensitivity in acute lymphocytic leukemia and induces mitochondrial permeability transition in breast cancer, facilitating mtDNA release.^289^

SIRT3, a mitochondrial deacetylase, plays a pivotal role in tumor metabolic regulation. Its function as a metabolic sensor is particularly prominent. In diffuse large B-cell lymphoma, SIRT3 drives glutamate transport into the TCA cycle, leading to nutrient deficiency. Through mitochondrial protein deacetylation, SIRT3 reverses the Warburg effect in lung cancer, regulates ROS levels in chronic lymphocytic leukemia, and participates in T-cell differentiation within the TME.^290–292^ Moreover, SIRT3 enhances ferroptosis by promoting mitochondrial autophagy in glioblastoma and disrupts fatty acid β-oxidation in the CSCs of AML.^293,294^

SIRT4, a uniquely functioning Sirtuin member, is involved in complex regulatory mechanisms in cancer biology. Its metabolic sensor function primarily manifests in the regulation of mitochondrial glutaminolytic metabolism. By inhibiting GDH, SIRT4 modulates mitochondrial glutaminolysis.^295^ In cervical cancer, SIRT4 interacts with the PIK3CA-E545K signaling axis to reduce radiotherapy sensitivity through glutamate metabolism regulation.^296^ In pancreatic cancer, SIRT4 can also enhance stemness through calcium signaling-stimulated histone lactylation and epigenetic reprogramming of metabolic pathways.^297^

SIRT5, a crucial mitochondrial deacetylase, influences tumor metabolism and progression by regulating protein posttranslational modifications such as desuccinylation, demalonylation, and deglutarylation.^298^ In colorectal cancer, SIRT5 promotes tumorigenesis by enhancing glutaminolytic metabolism.^299^ SIRT5 can regulate key mitochondrial metabolic enzymes such as malic enzyme 2 (ME2) and SHMT2 through desuccinylation, promoting tumor cell proliferation.^300,301^ In AML, SIRT5 is critical for maintaining mitochondrial OXPHOS, preserving redox balance, and driving glutaminolysis.^302^

These sirtuin family members are not merely passive metabolic regulators but also active modulators of tumor progression. Through complex molecular mechanisms, they integrate multidimensional cellular signals, providing new theoretical foundations and potential targets for precision cancer therapy.

### Organelle coordination

Mitochondria rarely function as isolated entities; instead, they establish extensive interactions with other organelles to coordinate critical metabolic and signaling functions. In cancer cells, metabolic flexibility and stress resilience are crucial for survival and proliferation. Interactions between mitochondria, the ER, peroxisomes, and the nucleus undergo significant reorganization to sustain tumor progression.

#### Mitochondria-ER cross talk

The functional and structural coupling between mitochondria and the ER is largely organized through mitochondria-associated membranes (MAMs).^303^ MAMs are not mere connecting regions between organelles but complex signal transduction and metabolic regulation centers. They precisely control calcium ion and bioactive lipid transport through key bridging proteins such as IP3 receptors (IP3Rs), VDAC1, and GRP75.^303^ In cancer cells, the upregulation or posttranslational modification of these components can increase mitochondrial calcium transport, activate prosurvival pathways (e.g., PI3K/Akt), and reduce apoptotic signal sensitivity.^304^ Missiroli et al. discovered the intricate P2X7 receptor-NLRP3 inflammasome trimeric complex and its fine-tuning by the tumor suppressor PML.^305^ Myoferlin, a new MAM, further enhances the system’s functional complexity by regulating mitochondrial calcium levels in pancreatic ductal adenocarcinoma cells.^306^ HK2 localization in MAMs reveals a new dimension of metabolic enzyme involvement in transmembrane signal regulation. The mitochondrial LON protein stabilizes the FUNDC1-ULK1 complex, regulating mitochondrial autophagy and participating in cell survival and cancer progression.^307^ Lipid exchange at MAMs facilitates rapid phospholipid and cholesterol transfer, which is crucial for membrane biosynthesis in highly proliferative cancer cells.^308^ Even under nutrient deprivation conditions, oncogenic remodeling of MAM-associated protein networks can enhance mitochondrial metabolism. Thus, targeting MAM components represents a viable strategy to disrupt cancer cell metabolism and reduce resistance to apoptosis.

#### Mitochondria-peroxisome interactions

Mitochondria and peroxisomes, as cellular metabolic hubs, play critical roles in cell survival and death regulation. These organelles collaborate in fatty acid oxidation and ROS level maintenance. Peroxisomes catalyze long-chain fatty acid oxidation through acyl-CoA oxidase, generating medium-chain acyl-CoAs and hydrogen peroxide as critical metabolic substrates and redox signals.^309^ Medium-chain acyl-CoAs are subsequently transported to mitochondria, oxidized through β-oxidation, and generate ATP, effectively supporting cancer cell metabolic reprogramming and sustained growth.^310^ Lee et al. used whole-genome CRISPR screening to reveal the importance of peroxisome, Golgi, and ER genes in mitochondrial biogenesis stress.^311^ Wilhelm et al. elucidated the molecular regulation of organelle-selective autophagy through BNIP3L/NIX protein mechanisms.^312^ Under hypoxic or iron-chelating conditions, BNIP3L can simultaneously localize to mitochondria and peroxisomes, coordinating autophagy through ATG8 family protein interactions. The critical role of the mitochondrial outer membrane E3 ubiquitin ligase MARCH5 in pre-peroxisome generation further reveals the molecular mechanisms of organelle dynamic remodeling.^313^ Phosphatidylinositol-5-phosphate 4-kinases (PI5P4Ks) play crucial roles in maintaining cellular energy homeostasis by regulating lipid transport and β-oxidation between peroxisomes and mitochondria.^314^

#### Mitochondria-nucleus signaling

The dynamic communication network between mitochondria and the nucleus constitutes a key molecular regulatory mechanism for the cellular stress response.^315^ This bidirectional signal transmission system precisely regulates cellular stress responses through retrograde and anterograde communication modes. Mitochondrial dysfunction can trigger transcription factors such as ATF4 and NFAT, increasing the expression of stress adaptation-related genes such as those involved in amino acid metabolism and GSH biosynthesis, thereby increasing cancer cell resistance to adverse environments.^316^ Simultaneously, anterograde signals precisely control the ETC, TCA cycle enzymes, and ROS detoxification pathways by regulating nuclear-encoded mitochondrial protein expression.^317^ Desai et al. proposed a mechanism of mitochondria-nucleus contact site formation, providing a new molecular perspective.^315^ The mitochondrial retrograde response (MRR) serves as a molecular response mechanism to internal and external perturbations. Its signal transduction is regulated by ROS, calcium ions, ATP, and lipid and protein homeostasis, playing crucial roles in transcription factor-driven gene expression.^318^ Based on mitochondria-nucleus migration probe, ultrasensitive monitoring of cancer treatment efficacy is achieved through real-time detection of mtDNA damage-induced apoptosis.^319^ This precise molecular communication network reveals cellular adaptive mechanisms and provides in-depth molecular insights into cancer cell metabolic reprogramming.

### Intercellular mitochondrial signaling

Mitochondria play crucial roles in cell-cell communication, influencing cancer progression, metastasis, and treatment resistance. This complex communication network is mediated by mitochondrial transfer through tunneling nanotubes (TNTs), Extracellular vesicles (EVs), and other mechanisms, which are essential for maintaining metabolic plasticity and helping cancer cells survive.^320^

Mitochondrial intercellular transfer represents a key molecular adaptation mechanism, forming a complex network of metabolic information exchange.^321^ Through diverse pathways, including TNTs, cell fusion, and phagocytosis, mitochondria achieve precise cross-cellular transfer. TNTs, specialized cytoplasmic extensions, establish direct connections between adjacent cells. Their formation is regulated by multiple factors, particularly those that are prominent under cellular stress conditions. Mesenchymal stem cell-mediated mitochondrial transfer reveals the molecular essence of tumor metabolic reprogramming by enhancing OXPHOS in melanoma cells and increasing chemotherapy resistance in glioblastoma cells.^322^ The cross-cellular sharing of mtDNA mutations in tumor-infiltrating T cells further elucidates the molecular strategies of mitochondrial transfer in tumor immune evasion.^323^ Miro proteins, key members of the small GTPase family, play pivotal roles in mitochondrial movement and intercellular transfer, with mitochondrial transfer closely associated with tumor progression and treatment resistance in ovarian and breast cancers.^324^ In mouse melanoma models, *Miro1* gene knockout effectively inhibited tumor growth by blocking mitochondrial transfer from stromal cells to cancer cells, further validating the critical role of mitochondrial transfer.^325^ Adipose-derived stem cell mitochondria significantly enhance multidrug resistance in breast cancer cells by regulating OXPHOS metabolism.^326^ Under hypoxic or chemotherapy-induced stress conditions, mitochondrial transfer exhibits complex unidirectional or bidirectional transmission patterns. Mitochondrial transport from stromal to cancer cells not only restores the bioenergetic capacity of damaged cancer cells but also enhances their metabolic plasticity, enabling efficient adaptation to the dynamic TME.^327^

EVs serve as critical molecular messengers and play an extraordinarily complex and precise regulatory role in intercellular mitochondrial component exchange. Their multidimensional molecular mechanisms reveal adaptive survival strategies for cancer cells.^328^ These vesicles are not passive transport carriers but are active mediators of cellular metabolism and signal transduction. They carry diverse bioactive molecules, including metabolic enzymes, lipids, nucleic acids, complete mtDNA, and mitochondrial protein fragments, enabling precise long-distance communication within and beyond the TME.^329^ Mitochondrial components in EVs demonstrate functional plasticity by reprogramming recipient cell metabolism. When exposed to EVs carrying functional mtDNA, recipient cells can restore defective mitochondrial function, significantly enhancing energy production capabilities and providing critical molecular support for cancer proliferation and metastasis.^330^ Cancer cell-released EVs precisely shape the TME by regulating stromal and immune cell functions. Exosomal mitochondrial components can modulate macrophage and dendritic cell activity, creating an immunosuppressive microenvironment. They simultaneously form a “metabolic hijacking” network with CAFs, transforming stromal cells into nutritional suppliers for cancer cells.^331^ In the hypoxic TME, small EVs actively regulate mitochondrial dynamics, not only transferring mitochondria but also carrying mtDNA and key regulatory factors. In colorectal cancer, they transfer complete mitochondrial genomes to surrounding epithelial cells, promoting TGF-β1-mediated tumor progression.^332^ Mitochondria-derived vesicles (MDVs) play crucial roles in tumor progression. When classical mitochondrial autophagy is blocked, cancer cells maintain mitochondrial homeostasis by increasing MDV release and achieving mitochondrial transfer through various pathways, including TNTs and phagocytosis.^333^ At the immune regulation level, mtDNA release and EV-mediated transfer may promote tumor immune evasion through the STING-IFN signaling pathway and suppress immune responses by affecting T-cell function.^334^ This complex molecular network helps tumor cells acquire chemotherapy resistance and reshape energy metabolism, providing continuous momentum for tumor progression. These findings reveal the multidimensional regulatory mechanisms of mitochondrial transfer in cancer biology, offering promising molecular targets and strategies for precision cancer therapy.

## Mitochondrial regulation of the tumor microenvironment

**Fig. 5:**
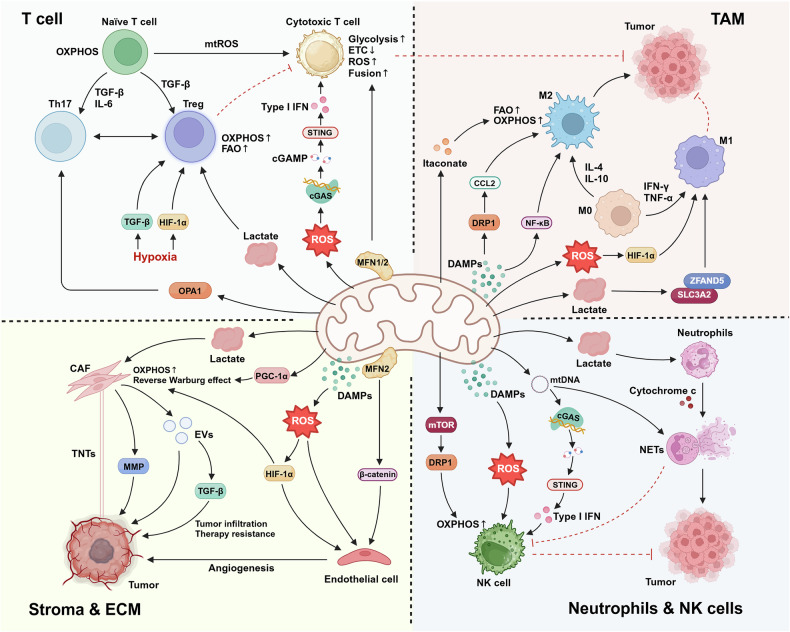
Mitochondrial regulation of cellular interactions in the tumor microenvironment. Mitochondria play crucial roles in coordinating metabolic and immune responses within the tumor microenvironment. Different immune cell types exhibit unique metabolic adaptations: T cells shift between oxidative phosphorylation and glycolysis, macrophages polarize between pro- and antitumor states through metabolic switching, and NK cells maintain antitumor activity via specific metabolic pathways. Cancer-associated fibroblasts support tumor metabolism by providing metabolic resources and signaling molecules. These intricate mitochondria-mediated interactions regulate tumor progression, immune responses, and potential therapy resistance, highlighting the complex metabolic landscape of cancer microenvironments. β-catenin catenin beta, CAFs cancer-associated fibroblasts, CCL2 C-C motif chemokine ligand 2, cGAMP cyclic GMP-AMP, cGAS cyclic GMP-AMP synthase, DAMPs damage-associated molecular patterns, DRP1 dynamin-related protein 1, ETC electron transport chain, EVs extracellular vesicles, FAO fatty acid oxidation, HIF-1α hypoxia-inducible factor 1-alpha, IFN-γ interferon-gamma, IL-4/10 interleukin 4/10, MFN1/2 mitofusin 1/2, MMPs matrix metalloproteinases, mtDNA mitochondrial DNA, mtROS mitochondrial reactive oxygen species, NETs neutrophil extracellular traps, NF-κB nuclear factor kappa B, NK natural killer, OPA1 optic atrophy 1, OXPHOS oxidative phosphorylation, PGC-1α peroxisome proliferator-activated receptor-gamma coactivator 1-alpha, STING stimulator of interferon genes, TGF-β transforming growth factor-beta, Th17 T helper 17, TNF-α tumor necrosis factor-alpha, TNTs tunneling nanotubes, Treg regulatory T cell, type I IFN type I interferon. This figure was created with BioRender (https://biorender.com/)

**Fig. 6:**
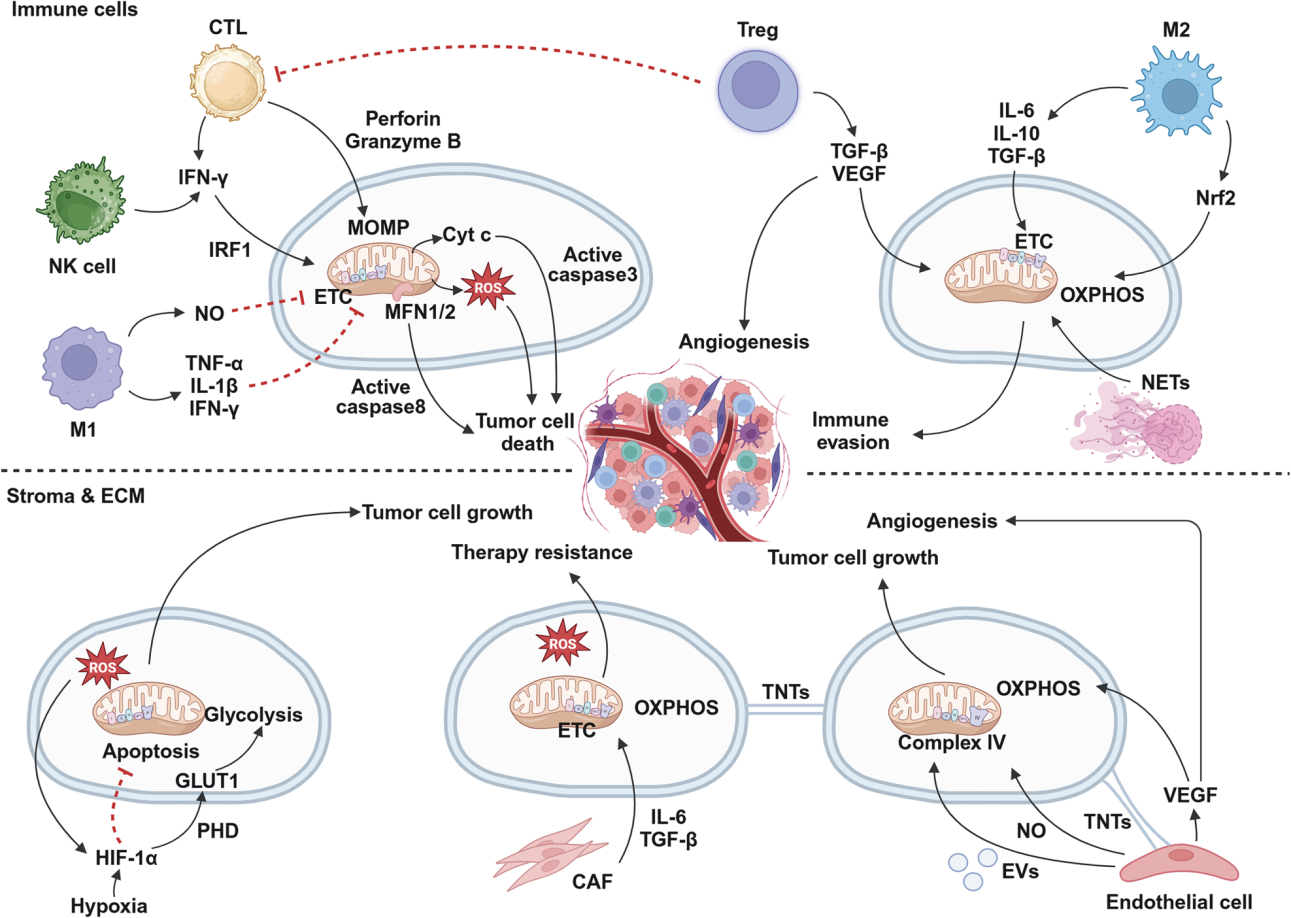
Mitochondria-mediated crosstalk in the tumor microenvironment. Mitochondrial interactions drive complex cellular dynamics within the tumor microenvironment. Immune cells such as cytotoxic T lymphocytes, natural killer cells, and M1 macrophages generate antitumor responses through reactive oxygen species and inflammatory signals. Conversely, immunosuppressive cells such as regulatory T cells and M2 macrophages promote tumor survival by secreting growth factors. Cancer-associated fibroblasts support tumor progression through metabolic resources and signaling molecules. Tumor cells adapt by modifying mitochondrial function, enhancing metabolic flexibility, and establishing intricate communication networks that ultimately facilitate tumor survival, angiogenesis, and resistance to therapeutic interventions. The mitochondria depicted in this figure are derived from cancer cells. CTLs cytotoxic T cells, CAFs cancer-associated fibroblasts, Cyt c cytochrome c, ETC electron transport chain, EVs extracellular vesicles, GLUT1 glucose transporter 1, HIF-1α hypoxia-inducible factor 1-alpha, IFN-γ interferon-gamma, IL-1β interleukin 1 beta, IL-6/10 interleukin 6/10, IRF1 interferon regulatory factor 1, MFN1/2 mitofusin 1/2, MOMP mitochondrial outer membrane permeabilization, NETs neutrophil extracellular traps, NO nitric oxide, Nrf2 nuclear factor erythroid 2-related factor 2, OXPHOS oxidative phosphorylation, PHD prolyl hydroxylase domain, ROS reactive oxygen species, TGF-β transforming growth factor-beta, TNTs tunneling nanotubes, TNF-α tumor necrosis factor-alpha, VEGF vascular endothelial growth factor. This figure was created with BioRender (https://biorender.com/)

Mitochondria are central to the dynamic interplay between cancer cells and the various immune populations that infiltrate the TME. While cancer cells often rewire their own mitochondria for increased proliferation and survival, these changes exert multifaceted influences on immune cell mitochondria, shaping both antitumor and immunosuppressive responses (Fig. 5). In turn, immune cells counteract these adaptations through metabolic activity, cytokine production, and direct cytotoxicity, potentially placing metabolic pressure on tumor mitochondria (Fig. 6). Notably, the precise mechanisms and overall impact of these mitochondrial interactions differ across tumor types, reflecting various metabolic dependencies, microenvironmental factors, and intrinsic biological features.

### Mitochondrial regulation of immune cells

#### T cells

##### Cytotoxic T lymphocytes (CTLs)

Mitochondria are critical orchestrators of CTL effector functions and play dual roles in energy production and intracellular signaling regulation. These organelles generate ATP to support robust proliferation and cytotoxicity while simultaneously modulating complex immunological responses.^335^

Mitochondrial-derived ROS are pivotal immunomodulators that facilitate mtDNA release, which can activate cGAS-STING signaling and increase interferon production. Conversely, metabolic alterations in the TME, such as elevated lactate levels, can directly suppress CTL activity, underscoring the delicate metabolic balance governing T-cell functionality.^336^ Mitochondrial protein dynamics play a crucial role in CTL performance. Heat shock protein 60 (HSP60) chaperone proteins positively regulate enzymes associated with glutaminolysis and fatty acid oxidation, thereby promoting intracellular α-ketoglutarate levels and mitochondrial ATP-dependent antiviral T-cell immune responses.^337^ Metabolic perturbations significantly impact CTL effector mechanisms. Glycolytic inhibition compromises F-actin remodeling and cell migration, whereas mitochondrial OXPHOS blockade restricts cellular extension during migration.^338^ Mitochondrial translation disruption triggers a decrease in cytosolic translation, impeding effector secretion and diminishing sustained cytotoxic capabilities.^339^ CTL functionality is intricately linked to mitochondrial integrity. Redox homeostasis disruption, compromised mitochondrial respiration, and dysfunction in calcium release-activated calcium (CRAC) channels can substantially impair immune responses. CRAC channel interference inhibits calcium influx, delaying degranulation and reducing target cell cytotoxicity.^340^

MFN1/2 are critical for maintaining OXPHOS and T-cell survival. ER-mitochondrion interactions, particularly MFN2-mediated processes, regulate calcium homeostasis and metabolic adaptation.^341^ Signaling pathways involving PERK-mediated eIF2α phosphorylation enhance autophagy and metabolic reprogramming, ultimately strengthening antitumor responses.^342^ The transcriptional coactivator PGC-1α has emerged as a potential therapeutic target, as its expression is capable of protecting CTLs from exhaustion.^343^ Sustained Akt signaling can downregulate PGC-1α, compromising T-cell mitochondrial capacity and impairing tumor-specific responses. These insights underscore the potential of metabolic interventions aimed at preserving mitochondrial fitness to optimize CTL-mediated tumor clearance.

While the intrinsic metabolic complexity of CTLs enables sophisticated immune responses, these cells also exert direct, targeted effects on cancer cell mitochondria through potent cytotoxic mechanisms.^344^ Upon recognition by cancer cells, CTLs release perforin and granzymes that disrupt the mitochondrial outer membrane, triggering apoptosis via cytochrome c release.^345^ Additionally, mitochondria-specific GPX4 inhibition enhances ferroptosis, promoting CTL infiltration and activation, thereby synergistically disrupting cancer cell mitochondrial integrity and strengthening antitumor immune responses.^346^

##### T helper (Th) cells

Mitochondria represent pivotal orchestrators of Th cell biology, transcending traditional energy production to emerge as sophisticated signaling platforms that dynamically regulate immune cell differentiation and function.^347^ While effector T cells conventionally rely on glycolysis for rapid ATP generation, diverse Th subsets leverage unique mitochondrial pathways to sustain complex immunological responses. The metabolic plasticity of Th cells is fundamentally governed by intricate signaling mechanisms. Upon T-cell receptor activation and CD28 costimulation, mitochondrial energy strategies facilitate the differentiation of CD4 T cells into specialized subsets. The Notch1 signaling pathway has emerged as a critical regulator, with its intracellular domain (N1ICD) modulating gene transcription under specific cytokine environments to guide Th1, Th2, Th17 and induced regulatory T-cell (iTreg) differentiation.^348^ TFEB, which is induced by nutritional stress or IL-2, plays a crucial role in CD4^+^ T-cell metabolism, with its loss in Treg cells compromising their accumulation and function in cancer models.^349^

Low-dose radiation exemplifies the precision of mitochondrial metabolic control by selectively enhancing tumor-infiltrating CD4^+^IFNγ^+^ Th1 populations through STING pathway stimulation without affecting CD8^+^ Tc1 cell or regulatory T-cell proportions.^350^ Th2 cell development is sensitive to mTOR pathway components, with Raptor-mTORC1-mediated enhanced OXPHOS facilitating the transition from quiescent to actively proliferating states.^351^

Tregs show remarkable metabolic adaptability and exhibit a profound dependence on OXPHOS and fatty acid oxidation, particularly in nutrient-depleted or lactate-rich TMEs.^352^ The YAP pathway enhances mitochondrial protein translation by upregulating Leucyl-tRNA synthetase 2 (Lars2), with nutritional interventions potentially modulating cellular function. Dietary leucine restriction combined with YAP inhibitors can induce mitochondrial dysfunction, ultimately suppressing tumor growth.^178^ Disruption of fatty acid-binding protein 5 (FABP5) triggers mtDNA release, activating cGAS-STING-IFN signaling and altering Treg suppressive capabilities.^353^

T helper 17 (Th17) cells demonstrate mitochondrial adaptations, characterized by elongated mitochondria with dense cristae that preserve OXPHOS in hypoxic environments.^177^ Fusion proteins such as OPA1 and regulators such as LKB1 maintain the mitochondrial structure and redox balance, sustaining inflammatory responses.^354^ Calcium signaling directly influences pathogenic Th17 cell inflammatory reactions through the regulation of mitochondrial function.^355^ SDH plays a crucial role in T-cell metabolic and epigenetic control, with its deficiency inducing proinflammatory gene expression and promoting Th1 and Th17 cell differentiation.^356^ GLUT3-dependent glucose uptake controls a metabolic-transcriptional circuit that governs Th17 cell pathogenicity.^357^

The intricate interplay between mitochondrial dynamics and Th cell function reveals a sophisticated regulatory network. Metabolic plasticity has emerged as a critical determinant of immune cell behavior, offering unprecedented insights into immune regulation and potential therapeutic interventions.

##### Memory T (Tm) cells

Tm cells exhibit a unique metabolic landscape characterized by distinctive mitochondrial adaptations that underpin their longevity and robust recall responses in the TME. Mitochondrial fusion configurations in Tm cells optimize ETC complex associations, enhancing OXPHOS and fatty acid oxidation, in contrast to effector T cells, where mitochondrial fission reduces ETC efficiency and promotes aerobic glycolysis.^358^ CD8^+^ Tm cells exhibit significant upregulation of cytosolic phosphoenolpyruvate carboxykinase (Pck1), and disruption of the Pck1-glycogen-pentose phosphate pathway compromises the GSH/GSSG ratio and elevates the ROS level, critically impacting Tm cell formation and maintenance.^359^ Notably, CD8^+^ T cells deficient in nuclear receptor subfamily 4 group A (NR4a) exhibit increased expression of stemness/memory-associated genes and robust mitochondrial OXPHOS capabilities.^360^ Mitochondrial pyruvate carrier (MPC) deletion promotes metabolic flexibility by generating acetyl-CoA through glutamine and fatty acid oxidation, enhancing pre-memory gene histone acetylation and chromatin accessibility, thereby driving CD8^+^ T-cell differentiation toward a memory phenotype. Intriguingly, CAR-T cells treated with MPC inhibitors demonstrate superior and more persistent antitumor activity.^361^ The mitochondrial fusion protein OPA1, which is crucial for crista biogenesis and respiratory function, plays an essential role in memory T-cell functionality.^362^ These metabolic changes become the hallmark of Tm cells, conferring remarkable adaptability across diverse microenvironmental conditions.

#### B cells

During B-cell activation, mitochondria serve as critical energy generators and biosynthetic platforms, predominantly fueling cellular processes through OXPHOS and fatty acid oxidation.^363^ Mitochondrial quality control pathways, including mitophagy, fission, fusion, and biogenesis, are essential for maintaining cellular integrity and immune responsiveness. Conditional knockout of *Tfam*, a key mitochondrial transcription factor, profoundly impacts B-cell development, blocking germinal center reactions and skewing B-cell differentiation toward memory- and senescence-associated phenotypes.^364^

Mitochondrial activity critically governs B-cell fate decisions. Elevated mitochondrial metabolism promotes class-switch recombination, whereas reduced activity favors plasma cell differentiation through ROS-mediated Bach2 regulation.^365^ Dynamic mitochondrial fusion, fission, and autophagy processes guide the trajectory of cellular differentiation. In the TME, B cells demonstrate remarkable metabolic plasticity, adapting to nutrient restrictions by prioritizing OXPHOS and utilizing retrograde signaling to regulate nuclear transcriptional programs.^315^

B-cell mitochondrial dysfunction has emerged as a significant factor in B-cell malignancies, suggesting potential therapeutic interventions. In chronic lymphocytic leukemia, malignant cells exhibit a metabolic shift toward OXPHOS,^366^ with strategies targeting SIRT3 or OXPHOS showing promising antitumor efficacy.^367^ Specific molecular interactions, such as LARS2^+^ B-cell regulation by dietary leucine and the Erbin-mitochondria axis, offer insights into cancer progression and potential immunotherapeutic targets.^368,369^

In addition to intrinsic metabolic regulation, B cells actively modulate tumor mitochondrial function through multiple mechanisms. They directly influence cancer cell mitochondria by secreting antibodies and cytokines that can alter the mitochondrial membrane potential.^370^ Cytokines such as IL-10 and TNF-α modify cancer cell mitochondrial pathways, whereas immunoregulatory mediators reshape the local immune microenvironment and metabolic landscape.^371^ This intricate interplay between B-cell mitochondrial dynamics and tumor metabolism represents a promising frontier for developing more sophisticated cancer immunotherapeutic strategies.

#### NK cells

NK cells navigate the nutrient-depleted TME through a sophisticated metabolic balancing act, dynamically shifting between glycolysis and mitochondrial OXPHOS to sustain cytotoxic functions.^372^ Amino acid metabolism, particularly arginine metabolism, has emerged as a critical determinant of NK cell performance. Arginine is essential for maintaining mitochondrial integrity and facilitating cellular expansion, with tumor-induced arginine depletion compromising mitochondrial efficiency and attenuating immune responses.^373^ Increased arginase activity in the TME further restricts NK cell-mediated killing by impairing mitochondrial homeostasis. Lipid metabolism, which is mediated by fatty acid oxidation and CPT1A, enhances mitochondrial function and supports sustained cytotoxic potential against tumors and viral threats.^374^

The hypoxic TME induces mitochondrial fragmentation in NK cells, compromising their antitumor functionality. This process, characterized by excessive mitochondrial division, leads to reduced metabolic capacity, increased ROS, and impaired granzyme B and IFN-γ expression, ultimately contributing to immune evasion and tumor progression.^372^ Recent therapeutic strategies target the mTOR-DRP1 axis to counteract metabolic suppression, with compounds such as asiaticoside showing promise in mitigating mitochondrial dysfunction through SMAD2 and mTOR/DRP1 signaling.^375^ Moreover, mitochondrial stress can also trigger mtDNA release, activating the cGAS-STING-IFN pathway, which paradoxically reinforces NK cell activity.^376^ Notably, mitochondrial dynamics play a crucial role in NK cell interactions with other immune cells, such as T cells and dendritic cells (DCs), where disrupted mitochondrial homeostasis can favor tumor immune evasion.^373,377^

NK cells orchestrate complex tumor immunosurveillance through multifaceted mitochondrial modulation mechanisms, dynamically altering cancer cell mitochondrial activation states to modulate cellular susceptibility to NK cell-mediated cytotoxicity.^378^ Mitochondrial binding of HK2 enhances hepatocellular carcinoma resistance to NK cell-induced lysis by reducing effector caspase 3/7 activity and inhibiting cytochrome c release without compromising NK cell activation.^379^ The secretion of cytokines, including IFN-γ and TNF-α, further disrupts cancer cell OXPHOS, induces mitochondrial stress, and reduces metabolic plasticity, ultimately enhancing immune-mediated cytotoxicity.^380^ This intricate interplay between NK cell mitochondrial dynamics and tumor metabolism represents a critical frontier in understanding and potentially manipulating antitumor immune responses.

#### Macrophages

Macrophages demonstrate metabolic flexibility, dynamically adapting energy utilization patterns in response to diverse activation signals and environmental cues. Proinflammatory macrophages (M1) characteristically exhibit elevated glycolysis and attenuated OXPHOS, suggesting that rapid ATP generation is critical for driving inflammatory responses.^381^ During M1 activation, isocitrate dehydrogenase 2 (IDH2) dynamically regulates the TCA cycle and mitochondrial energy output.^382^ Concurrently, mitochondrial arginase-2 enhances complex II activity, shaping succinate accumulation, HIF-1α stabilization, and IL-1β secretion, which collectively reinforce M1 polarization.^383^ In contrast, anti-inflammatory macrophages (M2) rely more extensively on sustained mitochondrial energy production, which is predominantly fueled by the β-oxidation of fatty acids.^384^ Manganese superoxide dismutase (MnSOD) further contributes to the M2 phenotype by mitigating oxidative stress and stabilizing metabolic homeostasis.^385^ Inflammatory M1 macrophages frequently exhibit augmented mitochondrial fission mediated by DRP1, which enhances proinflammatory capacity partially through TLR9-NF-κB-driven chemokine production.^386^

Metabolic plasticity involves complex signaling interactions. Disruption of the Hedgehog signaling pathway can drive macrophages to prioritize glycolysis over fatty acid oxidation, effectively shifting them from an M2 phenotype to an M1-like phenotype.^387^ Conversely, metabolic crosstalk with adipocytes enables mitochondrial transfer to energy-depleted macrophages, preserving oxidative metabolism and mitigating oxidative stress.^388^ Key metabolic intermediates derived from mitochondria orchestrate immune signaling. Succinate accumulation stabilizes HIF-1α and bolsters proinflammatory cytokine production,^389^ while itaconate inhibits IDH2, creating negative feedback that modulates inflammation and redox balance.^390^ Furthermore, arginine depletion in a specific TME can compromise macrophage mitochondrial function, polarizing cells toward an inflammatory phenotype and demonstrating the profound impact of metabolite-driven immunomodulation.^373^

Mitochondrial quality control mechanisms play crucial roles in the metabolic regulation of macrophages. PINK1/Parkin-dependent mitophagy can effectively eliminate damaged mitochondria, mitigating elevated ROS and preserving metabolic fitness.^391^ TFEB influences mitophagy via mTOR-mediated phosphorylation events, establishing a critical link between autophagic flux and M1 polarization.^392^ Disruption of these quality control pathways can precipitate aberrant mitochondrial swelling and calcium dysregulation, as observed with chenodeoxycholic acid (CDCA) exposure, leading to pathological lipid accumulation and impaired M2 maturation.^393^

Tumor-associated macrophages (TAMs) exert a profound influence on tumor progression through intricate metabolic interactions. M2-like macrophages, in particular, modulate tumor mitochondrial function via paracrine signaling and direct metabolic exchanges. Cytokines such as IL-6 and IL-10 secreted by M2 macrophages can activate key metabolic regulators such as STAT3 in cancer cells, promoting both glycolysis and OXPHOS.^394^ This metabolic reprogramming facilitates cancer cell proliferation and can confer resistance to chemotherapy and immunotherapy.^395^ High IL-6 levels frequently correlate with adverse clinical outcomes across multiple cancer types, underscoring the critical importance of macrophage-tumor mitochondrial crosstalk.^396^

#### Neutrophils

While it is traditionally believed that neutrophils rely predominantly on glycolysis, recent evidence indicates that mitochondrial OXPHOS can become critical under specific physiological and pathological conditions.^397^ In the hypoxic and nutrient-depleted TME, neutrophils undergo significant metabolic transformations to maintain effector functions. Under glucose-restricted conditions, neutrophils sustain oxidative stress and immunosuppressive capabilities through mitochondrial fatty acid oxidation and c-Kit signaling pathways.^398^ Protein deficiency and nutritional imbalances compromise neutrophil maturation by reducing ATP production and disrupting mitochondrial dynamics.^399^ Key regulatory pathways maintaining neutrophil metabolism include mTOR governing mitochondrial activation during chemotaxis and AMPK sensing energy status, with shifts between glycolysis and mitochondrial respiration during differentiation.^400^

Mitochondrial dynamics and protein interactions play crucial roles in neutrophil function. The mitochondrial protein Syntaphilin (SNPH) regulates ATP production and modulates interactions between OXPHOS and glycolysis, with SNPH deficiency increasing neutrophil motility through increased mitochondrial activity.^401^ Conversely, MFN2 deletion increases cytosolic calcium, activating calcium-dependent protein kinase II, thereby reducing cell adhesion and altering neutrophil migration.^402^ The balance between mitochondrial fission and fusion largely depends on proteins such as DRP1 and OPA1, which are the primary determinants of neutrophil behavior under metabolic constraints in the TME.^403^ Nicotinamide adenine dinucleotide phosphate oxidase complex 2 (NOX2) deficiency triggers pyroptotic cell death by increasing mitoROS, inhibiting AMPK activation, and activating Gasdermin E.^404^ MitoROS function as critical second messengers, activating transcription factors such as NF-κB and driving cytokine and chemokine secretion.^405^ However, excessive ROS can exacerbate local inflammation and damage other immune cells, inadvertently facilitating tumor immune escape.

Neutrophil extracellular traps (NETs) represent a complex interplay of mitochondrial metabolism and immune function. When composed of DNA, histones, and granular proteins, NETs originate not only from cell nuclei but also from mitochondria.^406^ Mitochondrial ROS serves as a critical mediator in NET formation, creating a bidirectional relationship where NETs both depend on and influence mitochondrial function in cancer progression.^407^ These structures can promote tumor cell proliferation and migration, modulate redox balance, trigger angiogenesis, and drive metabolic reprogramming. NETs can activate cancer cell Toll-like receptor 4 (TLR4) through neutrophil elastase release, upregulating PGC-1α to promote mitochondrial biogenesis and tumor growth.^408^ Tumor-associated senescent neutrophils (Naged, CXCR4^+^CD62L^low^) form unique mitochondria-dependent NETs to facilitate breast cancer lung metastasis. Tumor-secreted NAMPT induces Naged formation, with SIRT1 serving as a critical transcription factor maintaining the Naged lifecycle through mitochondrial autophagy.^409^ Respiratory chain-generated mitoROS are now recognized as crucial for NETosis, partly because of mtDNA release.^410^ These findings underscore the potential for targeted interventions that could modulate neutrophil metabolism to enhance antitumor immunity.

### Stromal cells

Stromal cells, including cancer-associated fibroblasts (CAFs), endothelial cells and adipocytes, dynamically exchange metabolites with tumor cells through mitochondrial reprogramming, promoting nutrient flux and sustaining cancer proliferation.

#### CAFs

CAFs exhibit remarkable metabolic heterogeneity, which is distinctly different from that of normal fibroblasts, particularly under glucose-deprived conditions. Mitochondrial calcium influx coordinates CAF growth and apoptosis during nutrient scarcity.^411^ Beyond elevated OXPHOS capabilities, CAFs demonstrate increased mitochondrial biogenesis mediated by critical signaling networks, including TGF-β, HIF-1α, and AMPK, which satisfy heightened ATP demands and facilitate extracellular matrix (ECM) remodeling.^412^ These pathways converge on transcriptional regulators such as PGC-1α, which orchestrates OXPHOS activity and global mitochondrial gene expression. Under aerobic conditions, CAFs frequently activate glycolysis, secreting lactate and pyruvate as part of a “reverse Warburg effect”, where adjacent cancer cells utilize these metabolic byproducts to fuel their TCA cycle and biosynthetic programs.^413^ This metabolic interdependence enhances cancer cell proliferation and viability, especially in hypoxic or nutrient-depleted TME regions.

Mitochondrial metabolic reprogramming in CAFs involves complex regulatory mechanisms that significantly contribute to tumor progression. The NF-κB pathway modulates glucose metabolism, providing energetic support for cancer progression.^414^ CAF diaphanous-related formin 3 (DIAPH3, mDia2) influences protein secretion, metabolic function, and ECM remodeling by stabilizing the MIRO1 protein and regulating mitochondrial localization and function.^415^ Moreover, multiple studies have highlighted the potential of G protein-coupled estrogen receptor (GPER) inhibition to induce CAF apoptosis through the JNK/c-Jun/p53/Noxa signaling axis, reducing CAF survival and consequently diminishing invasive tumor phenotypes by weakening fibroblast-derived paracrine factors.^416^

The intricate interactions between CAFs and cancer cells reveal sophisticated metabolic communication mechanisms. CAF-derived mitochondria can regulate lung cancer cell metabolism through the ROS and TGF-β signaling pathways.^417^ Intriguingly, cleaved type I collagen can initiate a DDR1-NF-κB-p62-NRF2 cascade, increasing mitochondrial biogenesis in CAFs and promoting more metastasis-conducive invasive matrix structures.^418^ Mitochondrial dynamics extend beyond internal cellular processes, with CAFs capable of directly transferring functional mitochondria to cancer cells via tunnel nanotubes, enhancing cancer cell bioenergetics and drug resistance.^419^ CAFs secrete ANGPTL4, which binds IQGAP1 to activate the Raf-MEK-ERK-PGC-1α axis, promoting mitochondrial biogenesis and OXPHOS metabolism to support prostate cancer cell growth and chemotherapeutic resistance.^420^

#### Endothelial cells

Tumor angiogenesis, which is mediated by endothelial cells, is critical for malignant growth and metastatic dissemination and provides essential oxygen, amino acids, and glucose to rapidly dividing cancer cells.^421^ Mitochondrial function plays a pivotal role in this process, with research demonstrating the profound impact of mitochondrial respiratory mechanisms on endothelial cell behavior. Deletion of the *Cox10* gene in mouse endothelial cells revealed the crucial importance of mitochondrial respiration in vascular development, wound healing, and tumor growth.^422^ Complex regulatory mechanisms govern mitochondrial function in endothelial cells, including the expression and functionality of mitochondrial complexes I-III, which directly influence cellular metabolism and proliferative capacity.^423^ Mitochondrial dynamics proteins, such as DRP1, are essential for maintaining endothelial cell function, with heparin improving mitochondrial morphology by inhibiting DRP1 mitochondrial translocation.^424^ The NOTCH1 intracellular domain (NICD1) can directly localize to mitochondria, enhancing metabolic capabilities by activating mitochondrial complexes and promoting endothelial-to-mesenchymal transition (EndMT).^425^ Recent single-cell RNA sequencing analysis by Lebas et al. revealed the pivotal role of mitochondrial calcium signaling in EndMT, demonstrating that the mitochondrial calcium uptake regulator MCU is crucial in this process, with MCU inhibition preventing EndMT.^426^ The ER transmembrane protein TMEM215 reduces mitochondria-ER membrane contact, decreases mitochondrial calcium intake and inhibits BIK-mediated apoptosis during vascular pruning.^427^

Specialized molecular mechanisms further modulate endothelial cell mitochondrial function and tumor angiogenesis. The SLC1A1 transporter facilitates R-2-hydroxyglutarate (R-2-HG) entry into cells and mitochondria, supporting endothelial cell migration by regulating mitochondrial respiration and sodium/calcium exchange.^428^ Mitochondrial NADP^+^-dependent IDH2 regulates OXPHOS complex expression and endothelial nitric oxide synthase (eNOS) phosphorylation, with IDH2 deficiency causing endothelial dysfunction.^429^ Inhibiting acid sphingomyelinase (ASM) increases intracellular ROS, triggering metabolic reprogramming, which includes reduced cell proliferation, decreased mitochondrial energy metabolism, and stimulated autophagy and antioxidant responses, ultimately promoting cerebrovascular generation while maintaining endothelial cell survival.^430^

#### Adipocytes

Adipocytes emerge as critical contributors to the TME, primarily through their capacity to store and release substantial lipids, including free fatty acids (FFAs), that can be readily absorbed by cancer cells.^431^ These FFAs support mitochondrial β-oxidation and OXPHOS, providing essential energy substrates under hypoxic or nutrient-depleted conditions.^432^ Lipid transfer involves key proteins such as CD36 and FABP4, highlighting the dynamic lipid exchange between adipocytes and cancer cells.^433^ EVs secreted by adipocytes can transport enzymes and fatty acids, significantly stimulating mitochondrial metabolic reprogramming in cancer cells.^434^ By transferring FFAs to cancer cells, adipocytes activate AMPK, induce mitochondrial fatty acid β-oxidation and autophagy, and promote EMT, substantially enhancing tumor growth and survival.^435^ This intricate metabolic crosstalk underscores the multifaceted role of adipocytes in cancer progression, transforming them from passive energy storage cells to active participants in metabolic adaptation and survival strategies in tumors.

## Mitochondrial contribution to therapy resistance

Mitochondrial adaptability is crucial for cancer cell survival under therapy-induced stress. Mounting evidence suggests that cancer cells leverage mitochondrial function to regulate intracellular ROS, reprogram metabolic pathways, and modulate apoptosis and immune responses. These processes not only sustain oncogenic signaling and protect critical cellular components but also reshape the TME to counteract various therapeutic strategies, ultimately promoting treatment resistance.

### Radiotherapy resistance

Radiotherapy eliminates cancer cells by inducing DSBs,^436^ whereas mitochondria mediate radioresistance through multiple mechanisms. Ionizing radiation (IR) not only directly damages nuclear DNA but also significantly increases mitochondrial respiratory chain protein activity, promoting ATP generation and ROS release, triggering secondary organelle damage and delaying DSB repair.^437^ Mitochondria contribute to radiation resistance by maintaining genomic stability, regulating redox balance, and supporting the energy supply. For example, post-radiation cancer cells suppress DNA damage sensing by hijacking caspase-9 signaling, whereas tumor-derived mtDNA can activate intrinsic DNA-sensing pathways.^438^ Notably, mtDNA is particularly susceptible to radiation-induced DSBs and point mutations because of its proximity to the ETC region.^439^ Mitochondrial complex I deficiency promotes DNA repair and enhances radioresistance by downregulating PDH.^440^ Inhibiting mitochondrial RNA polymerase (POLRMT) reduces mtDNA content and respiratory chain complex levels, suppressing NSCLC growth.^441^ Moreover, autophagy gene deletion enhances radiotherapy sensitivity by releasing mtDNA and type I interferons, activating antitumor immunity.^442^ PNKP deficiency causes mtDNA damage, the accumulation of ROS, and the triggering of cytoplasmic DNA accumulation and STAT1 phosphorylation.^443^ These findings suggest that the dynamic balance between mitochondrial metabolism and DNA repair is a critical determinant of the response to radiotherapy.

Mitochondria also regulate radioresistance by integrating multidimensional signaling networks. In hepatocellular carcinoma, nuclear paraspeckle assembly transcript 1 (NEAT1) variant 1 (NEAT1v1) promotes mitochondrial autophagy and reduces oxidative stress by increasing the expression of γ-aminobutyric acid A receptor-associated protein (GABARAP) and SOD2, increasing radioresistance.^444^ In glioma, DNA-dependent protein kinase phosphorylates PGC-1α, facilitating its ubiquitination by RNF34 and suppressing mitochondrial biogenesis to maintain radioresistance.^445^ Radiation-induced COX-2 upregulates TFAM expression through the p38-MAPK/DRP1 signaling axis, reducing cellular radiation sensitivity.^446^ Conversely, TFAM reduction exacerbates mitochondrial superoxide accumulation and DNA damage by enhancing the p53-MDM2 interaction.^447^ Prohibitin maintains low ROS levels by binding to PRDX3, promoting cancer stem cell self-renewal and radioresistance.^448^ SIRT3 enhances DNA repair through PINK1/Parkin-mediated autophagy,^449^ whereas SGLT1 regulates post-radiation mitochondrial autophagy via the PI3K/AKT/mTOR pathway.^450^ Additionally, TRIM21 weakens radiation-induced immune responses by inhibiting VDAC2 oligomerization and mtDNA release,^451^ and X-ray-induced incomplete MOMP drives radioresistance through eIF2α/ATF4 signaling.^452^ These mechanisms reveal how mitochondria achieve adaptive radiation remodeling through epigenetic regulation, protein interactions, and organelle crosstalk.

### Chemotherapy resistance

The core mechanism of chemotherapy resistance involves adaptive mitochondrial functional remodeling in cancer cells. Traditional chemotherapeutic agents target DNA replication or spindle function to eliminate rapidly proliferating cancer cells. However, cancer cells can escape cytotoxicity through mitochondrial metabolic reprogramming.^453^ For example, abnormal activation of mitochondrial OXPHOS promotes drug resistance by maintaining tumor stemness. In bladder cancer, the mitochondrial transcriptional activator TACO1 enhances OXPHOS activity and increases mtROS by promoting mitochondrial cytochrome oxidase I (MTCO1) translation, with its mitochondrial localization regulated by HSP90β and circFOXK2.^454^ Simultaneously, mitochondria determine the apoptotic fate by regulating outer membrane permeabilization (MOMP). BAK/BAX activation can induce MOMP and initiate caspase cascade reactions,^455^ whereas BCL-2 family protein mutations inhibit MOMP.^456^ Notably, MOMP has dual effects: when caspase-3/7 is suppressed, released mtRNA triggers inflammation through the MDA5/MAVS/IRF3 pathway,^345^ whereas NF-κB pathway activation may generate antitumor effects by downregulating antiapoptotic proteins.^457^ This dynamic balance suggests that targeting apoptosis regulation requires the integration of microenvironmental signal networks.

Abnormal mitochondrial dynamics and noncoding RNAs also participate in the regulation of resistance to chemotherapy. Excessive fission mediated by DRP1 promotes drug resistance in breast, liver, pancreatic, and colon cancers,^458^ whereas osteosarcoma stem cells maintain mitochondrial morphological stability and chemotherapeutic resistance through DRP1.^459^ Conversely, ELK3 reduces triple-negative breast cancer sensitivity to cisplatin by inhibiting fission and ROS generation,^460^ and DNA damage agents enhance TCA cycle metabolic adaptability through OPA1-mediated mitochondrial fusion.^461^ At the epigenetic level, METTL3 promotes small cell lung cancer resistance by inducing mitochondrial autophagy through m^6^A methylation,^462^ and FTO alters mitochondrial dynamics through CDKAL1 mRNA demethylation.^463^ Notably, disruption of the p-p53(Ser15)-Phb1-Bak complex directly causes mitochondrial homeostasis imbalance,^464^ whereas glioblastoma stem cells maintain stemness by regulating the NAD^+^/NADH ratio through CYP3A5,^465^ highlighting the intersection of metabolism and epigenetic regulation.

Current research reveals the complexity and compensatory potential of mitochondrial networks. While targeting OXPHOS, MOMP regulation, or key dynamic proteins (such as DRP1 and OPA1) shows therapeutic promise, interactions between mitochondria and ER/lysosomes and metabolic-epigenetic coupling mechanisms may increase intervention challenges.

### Immunotherapy resistance

Tumor resistance to immunotherapy involves a complex interplay between mitochondrial dysfunction and immune evasion mechanisms. This network can be summarized by three core processes: metabolic competition reshaping the TME, imbalances in mitochondrial dynamics that mediate immune signal disruption, and epigenetic modifications that regulate immune checkpoint expression. Immunotherapy aims to activate the immune system against cancer cells,^466^ whereas mitochondrial oxidative stress, fragmentation, and DNA leakage directly trigger immune escape.

By enhancing glycolysis and adaptive mitochondrial metabolism, cancer cells deplete glucose, glutamine, and oxygen in the TME, leading to exhaustion of T-cell mitochondria.^467^ High glycolytic phenotypes disrupt T-cell Ca^2+^-NFAT signaling by depleting glucose and phosphoenolpyruvate, thus suppressing interferon-γ production.^468^ Lactate accumulation acidifies the microenvironment, impairing T-cell proliferation and cytolytic functions.^469^ Under hypoxic conditions, persistent antigen stimulation inhibits T-cell PGC-1α-dependent mitochondrial reprogramming, triggering ROS accumulation and metabolic failure.^470^ Similarly, mitochondrial fragmentation in NK cells leads to a loss of tumor-killing capacity.^372^ This metabolic imbalance results in a malignant cycle with abnormal PD-1/PD-L1 axis activation. PD-1 signaling upregulates CPT1A, promoting T-cell fatty acid oxidation, inhibiting differentiation, and disrupting the mitochondrial crista structure.^471^ Overexpressed FASN suppresses PD-L1 modification and OXPHOS, weakening T-cell responses.^472^ Notably, mitochondrial damage and immune checkpoint expression form a positive feedback loop. In melanoma, mtDNA induces PD-L1 expression through the STING-IFN pathway,^334^ whereas ACLY inhibition-induced mtDNA leakage similarly upregulates PD-L1 and promotes T-cell exhaustion.^473^

Mitochondrial autophagy, epigenetic reprogramming, and mitochondrial hijacking further exacerbate immune resistance. Single-cell sequencing revealed that inhibiting mitochondrial autophagy reduces CAR-T-cell antitumor capabilities,^474^ whereas hypoxia-induced autophagy weakens antigen presentation by downregulating MHC-I.^475^ NK cell autophagy defects cause impaired mitochondrial polarization, but autophagy activation can restore functionality,^476^ suggesting spatiotemporally specific autophagy regulation. At the epigenetic level, IDH2-mutated gliomas escape NK cell killing through NKG2D ligand hypermethylation.^477^ ACLY inhibition-induced polyunsaturated fatty acid peroxidation induces PD-L1 expression through epigenetic modifications.^473^ Critically, cancer cells hijack T-cell mitochondria via nanotubes,^478^ with PD-1 overexpression enhancing this process,^479^ creating a “metabolic predation-immune paralysis” cycle. Furthermore, PD-1 signaling in CD8^+^ TILs contributes to reduced mitochondrial fitness through impaired mitophagy, driving epigenetic reprogramming toward terminal exhaustion,^480^ and mtDNA release, while activating the STING pathway, unexpectedly induces PD-L1 upregulation,^334^ highlighting the pathological coupling between mitochondrial stress and immune checkpoint regulation.

In summary, these findings collectively underscore mitochondria as a critical regulatory nexus in modulating cancer cell therapy resistance, revealing their multifaceted role in orchestrating cellular survival mechanisms under therapeutic stress.

## Mitochondrial-targeted tumor treatment strategies

### OXPHOS inhibitors

Triphenylphosphonium cation (TPP^+^)-conjugated drugs that target the mitochondrial ETC have demonstrated multidimensional antitumor mechanisms in recent basic research. MS-L6 selectively inhibits complex I and uncouples OXPHOS, inducing metabolic collapse in cancer cells. Its ability to achieve tumor-selective killing in vitro and in mouse models highlights the unique advantages of mitochondrial-targeted strategies.^481^ IACS-010759 not only suppresses proliferation in brain cancer and AML models but also induces oxidative stress, depleting reduced GSH, a mechanism particularly significant in MYC-overexpressing cells.^482^ Notably, the high sensitivity of SMARCA4-deficient renal cancer to IACS-010759 reveals the intrinsic correlation between the genetic background of the tumor and OXPHOS inhibitor efficacy.^483^ The impact of the dual-specificity tyrosine phosphorylation-regulated kinase 1A (DYRK1A)-regulated TGF-β signaling pathway on hepatocellular carcinoma resistance provides a theoretical basis for combination targeted therapy.^484^ The discovery of nonapoptotic death mechanisms further expands the dimensions of drug action. VLX600 achieves antitumor effects through autophagy-dependent death,^485^ whereas HP661 overcomes MEK inhibitor resistance and demonstrates specificity against high-OXPHOS-phenotype tumors.^486^ These findings collectively outline the potential of TPP^+^ drugs to reshape the TME through energy metabolism intervention.

However, clinical translation faces significant challenges. High-activity inhibitors such as IACS-010759 and BAY87-2243 encountered dose-limiting toxicities during dose-escalation trials, including metabolic acidosis and neurotoxicity, resulting in multiple clinical trial terminations due to narrow therapeutic windows.^487^ ASP4132 remains tolerable at low doses but results in severe adverse reactions above 5 mg,^488^ whereas the stable disease rate of IM156 suggests that the efficacy assessment criteria should be reevaluated.^489^ Interestingly, moderate-intensity inhibitors have unique advantages. Traditional drugs such as metformin improve tumor reoxygenation through radiosensitization in preclinical models,^490^ with atovaquone combination radiotherapy trials preliminarily validating this strategy (NCT04648033). Ongoing phase I trials will provide new insights into mitochondrial-targeted strategies (NCT04827810). ME-344 combined with bevacizumab, while not achieving an objective response, has a 39% disease control rate, suggesting its potential for clinical development in refractory colorectal cancer.^491^

Current research reveals a critical paradox. Although high-activity OXPHOS inhibitors excel in mechanistic studies, their dose-response curves result in narrow clinical windows. Conversely, traditional less effective drugs can achieve safe and effective tumor control through metabolic regulation or combination therapies. This contradiction suggests that future research should balance drug potency with systemic toxicity while strengthening biomarker research for precision medicine.

### IDH inhibitors

IDH enzymes exist in the cytoplasm (IDH1) and mitochondria (IDH2 and IDH3), catalyzing irreversible oxidative decarboxylation of isocitrate to α-ketoglutarate and NADPH in mitochondria. They play crucial roles in gene regulation, cell differentiation, and tissue homeostasis.^492^ Mutant IDH abnormally accumulates D-2-hydroxyglutarate (D-2-HG), triggering epigenetic reprogramming and blockade of cell differentiation, which are particularly prominent in gliomas and hematological malignancies.^492^ Selective mutant IDH inhibitors such as ivosidenib and vorasidenib reshape tumor epigenomes by reducing D-2-HG levels. Early clinical trials not only induced a reduction in glioma volume but also revealed deeper mechanisms of astrocyte differentiation and cancer stem cell pool reduction through single-cell sequencing.^493^ Vorasidenib’s breakthrough in the INDIGO phase III trial extended median progression-free survival from 11.1 to 27.7 months in IDH-mutated glioma patients, significantly delaying secondary intervention, despite 9.6% ≥grade 3 liver enzyme elevation.^494^ In patients with IDH1-mutated myeloid malignancies, the combination of ivosidenib and venetoclax increased composite complete remission rates to 90%, with 64% of long-term patients achieving IDH1 mutation clearance, suggesting the potential for deep molecular remission.^495^

Current clinical evidence reveals the disease-specific efficacy of IDH inhibitors. Gliomas demonstrate tumor growth arrest and differentiation induction, whereas hematological malignancies show molecular remission and survival benefits. This difference may stem from varying D-2-HG biological effects across tissue microenvironments, with gliomas’ immune-privileged characteristics potentially weakening immune-mediated clearance.^496^ Systematic analysis revealed that IDH inhibitors significantly improved progression-free survival (HR = 0.39) and disease control rates but did not significantly affect overall survival. These findings suggest that monotherapy may primarily control disease rather than provide a definitive cure. Preclinical NOTCH1 mutation resistance mechanisms and adverse reactions such as hepatotoxicity constitute current therapeutic bottlenecks, urgently necessitating predictive biomarker development and combination therapy strategies.^493,494^

### Glutaminase inhibitors

Glutamine metabolism serves as a critical hub maintaining cancer cell mitochondrial function by providing carbon scaffolds and energy precursors, driving OXPHOS and biosynthesis.^497^ Research has demonstrated that glutamic-oxaloacetic transaminase 2 (GOT2) knockout promotes hepatocellular carcinoma progression by activating the PI3K/AKT/mTOR pathway, simultaneously increasing sensitivity to the glutaminase inhibitor CB-839.^498^ TP53 mutations induce metabolic reprogramming in prostate cancer by upregulating asparagine synthetase (ASNS), with glutaminase inhibitors capable of synergistically blocking asparagine biosynthesis.^499^ CB-839 not only suppresses energy metabolism in KRAS-mutated lung cancer, enhancing radiotherapy sensitivity,^500^ but also integrates with photothermal-responsive hydrogels for spatiotemporally controlled drug release, improving radiotherapy by inhibiting DNA repair pathways.^501^ In combination with 5-FU, CB-839 induces NET formation and triggers apoptosis via protease G, demonstrating specific efficacy in PIK3CA-mutated colorectal cancer.^502^ BPTES, another glutaminase inhibitor, synergizes with HDAC inhibitors to target multiple leukemia stem cell survival signals, highlighting metabolic intervention and the intersectional effects of epigenetic regulation.^503^

Clinical translation remains challenging. CB-839 combined with capecitabine shows good tolerability in PIK3CA-mutated colorectal cancer, with Nrf2-UPP1 axis activation providing biomarker development insights.^504^ In metastatic renal cell carcinoma, telaglenastat combined with everolimus or cabozantinib achieves 95%-100% disease control without dose-limiting toxicity.^505^ However, phase III trials revealed a median progression-free survival of only 3.8 months, with 74% grade 3-4 adverse events, reflecting the complex balance between efficacy and toxicity.^506^ More promisingly, telaglenastat combined with azacitidine achieves complete remission in 53% of myelodysplastic syndrome patients. Additionally, glutamine transporter expression may predict treatment response, potentially opening new precision metabolic therapy avenues.^507^

### Mitochondrial apoptosis induction

Small-molecule strategies targeting mitochondrial apoptosis pathways provide multidimensional interventions by regulating the mitochondrial membrane potential, cytochrome c release, and BCL-2 family protein balance. Recent innovations include quinazoline-2-indolinone derivatives that activate mitochondrial apoptosis by inhibiting PI3Kα,^508^ whereas Cy5-I-CF3 enhances immunogenic cell death by synergistically inducing ER stress and mitochondrial apoptosis.^509^ Notably, CO/NO codelivery gas systems demonstrate unique mitochondrial targeting, inducing mPTP opening and cytochrome c release for efficient antitumor activity.^510^ Mechanistically, ponicidin stabilizes the Keap1-PGAM5 complex, triggering mitochondrial damage and ROS accumulation.^511^

BH3 mimetic clinical applications and resistance mechanism analysis represent significant advances. Venetoclax, an FDA-approved BCL-2 inhibitor, activates MOMP and caspase cascades.^512^ Resistance mechanisms involve MCL-1 overexpression and disruption of mitochondrial calcium homeostasis,^513^ driving dual-targeting strategies. Combining BCL-2 and PI3K inhibition significantly enhances AML cell apoptosis sensitivity.^514^ Recent CRISPR screening identified COX4I1 deletion as a potential sensitization mechanism for venetoclax combination therapies.^515^ Innovative combinations include venetoclax with actinomycin D for epigenetic-mediated AML cell elimination,^516^ and the GPER agonist G-1 for simultaneous apoptosis, pyroptosis, and antitumor immunity activation.^517^

### ROS inducers

Tumor ROS-targeting strategies exploit the vulnerable redox homeostasis of cancer cells. Malignant tumors exhibit elevated baseline ROS levels and compromised antioxidant defenses due to rapid proliferation, providing a molecular window for selective targeting.^518^ Recent developments have focused on tumor-specific ROS induction. Cinnamic aldehyde and PEG-based polymers such as ECPCP nanoparticles release copper ions in high-ROS TMEs, achieving precise killing through copper death and immunogenic cell death.^519^ Similar approaches include NQO1-responsive nanocarriers that amplify ROS through enzymatic cascades while reducing systemic toxicity.^520^ Lysosome-targeted nanocarriers such as VC@^N3AM^ cLAVs catalyze H₂O₂ conversion to hydroxyl radicals, triggering ferroptosis.^521^ Pyrrolidine-NO donor hybrid molecules induce prostate cancer cell apoptosis through combined NO release and ROS/DNA damage.^522^

Combination strategies enhance ROS sensitivity through metabolic intervention. FASN inhibitors increase membrane lipid unsaturation, sensitizing melanoma to arsenic trioxide and improving survival rates to 72% in mouse models.^225^ The NQO1-dependent ROS inducer napabucasin uses convection-enhanced delivery to overcome the blood-brain barrier, significantly improving local control of glioma when combined with radiotherapy.^523^ ROS inducers such as piperlongumine can reshape oxidative stress networks in glioblastoma, reversing radiotherapy/temozolomide-induced ROS depletion and restoring apoptosis induction.^524^ The mesenchymal phenotype resistance of NRAS-mutated melanoma can be effectively targeted by ROS inducers combined with MEK inhibitors.^525^ QD394 triggers ferroptosis by suppressing the STAT3-GPX4 axis, suggesting a new therapeutic paradigm for pancreatic cancer.^526^

### Integrated dynamics modulation

Targeting mitochondrial fission strategies offers new perspectives for interventions in tumor metabolic plasticity. Abnormal mitochondrial dynamics in cancer cells often manifest as excessive fragmentation. Inhibiting DRP1-mediated fission not only suppresses cancer stem cell characteristics by prolonging mitochondrial networks (reducing CD44 and OCT4 expression) but also triggers lipid peroxidation-dependent ferroptosis.^527^ Although early studies considered mdivi-1 a DRP1 inhibitor, its mechanism is more complex. At 50 μM, mdivi-1 reversibly inhibits mitochondrial complex I activity and regulates ROS generation,^528^ while enhancing paclitaxel-induced cell cycle arrest by binding to microtubulins.^529^ Glutamine deficiency activates DRP1 phosphorylation through the MAPK-ERK1/2 pathway, promoting mitochondrial fragmentation and cancer stem cell phenotypes. Combining L-DON with mdivi-1 can synergistically reverse this resistance mechanism.^530^ Deeper regulatory networks involve SUMOylation modifications. The SYNJ2BP-COX16 fusion protein promotes mitochondrial fission by enhancing DRP1 SUMOylation, a process that is blocked by mdivi-1.^531^ These findings suggest that the efficacy of mitochondrial fission inhibitors depends not only on direct DRP1 targeting but also on intersectional metabolic reprogramming and epigenetic modifications.

Mitochondrial biogenesis intervention strategies demonstrate diversification. As a core mitochondrial biogenesis hub, PGC-1α integrates upstream signals to activate mitochondrial genome programs, driving metabolic reprogramming.^532^ Genetically engineered PGC-1α can enhance CAR-T-cell metabolic adaptability and antitumor persistence in the TME.^532^ Notably, the p53 codon 72 Pro variant activates mitochondrial metabolism through PSAT1-mediated PGC-1α nuclear translocation. Its inhibitor aminooxyacetic acid (AOA) combined with regorafenib can specifically block this pathway and suppress hepatocellular carcinoma metastasis.^533^ The HPV E6 protein weakens mitochondrial biogenesis by suppressing the PGC-1α/ERRα axis,^534^ whereas MYC inhibition and PGC-1α upregulation can selectively target OXPHOS-dependent CSCs.^535^ Benorilate restores mtDNA content by activating downstream HIF-1α signals, reversing tamoxifen resistance.^536^ CD47 antibody-engineered oncolytic viruses (oAd-αCD47) combined with NaBi enhance immune cell mitochondrial metabolism by activating the CaMKII/CREB/PGC-1α axis.^537^ The “right-side-out” RBCm-camouflaged cationic micelle system precisely inhibits biogenesis to improve triple-negative breast cancer therapy.^538^

### Mitochondrial genome targeting

Abnormal mtDNA amplification is closely correlated with malignant tumor progression. In KRASG12D-driven lung tumors, increased mtDNA copy number directly enhances mitochondrial respiration. Transgenic mouse models have shown that mtDNA amplification significantly increases the tumor burden, whereas mtDNA depletion inhibits growth, independent of immune microenvironment changes.^539^ Intervention strategies include genetic tools and pharmacological approaches. MitoTALEN targets pathogenic mtDNA mutations to restore OXPHOS function.^540^ The nucleoside analog 2’3’-dideoxycytidine (ddC) selectively inhibits DNA polymerase γ, eliminating AML leukemia stem cells by blocking mtDNA replication.^541^ Alovudine drives breast cancer cell metabolism toward glycolysis by suppressing mtDNA synthesis. While showing limited primary tumor suppression (~20%), it reduces metastatic lesion formation by nearly 70%, highlighting high mtDNA content as a critical driver of metastasis.^542^

**Fig. 7:**
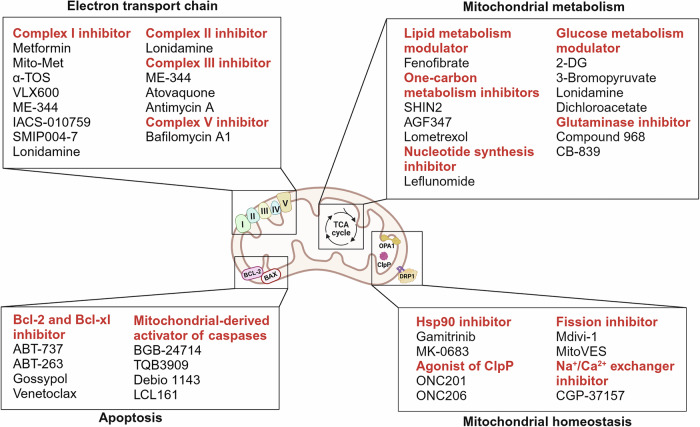
Representative mitochondrial therapeutic targets and their modulators. The figure illustrates four major intervention areas in mitochondrial-targeted therapy: (1) electron transport chain inhibitors, which target complexes I-IV; (2) mitochondrial metabolism modulators, which include regulators of lipid metabolism, glucose metabolism, one-carbon metabolism, and glutamine metabolism; (3) apoptosis pathway modulators, which include Bcl-2/Bcl-xl inhibitors and mitochondrial-derived activators of caspases; and (4) mitochondrial homeostasis modulators, which include Hsp90 inhibitors, ClpP agonists, fission modulators, and Na^+^/Ca^2+^ exchanger modulators. BAX BCL-2-associated X protein, BCL-2 B-cell lymphoma 2, ClpP caseinolytic peptidase P, DRP dynamin-related protein 1, OPA1 optic atrophy 1, TCA cycle tricarboxylic acid cycle. This figure was created with BioRender (https://biorender.com/)

In summary, mitochondrial-targeted cancer therapies reshape metabolic microenvironments by intervening in OXPHOS, IDH mutation, and glutamine metabolism. Despite their significant potential in overcoming drug resistance, clinical translation faces challenges related to balancing dose toxicity and efficacy. Various mitochondrion-targeted therapies are currently being evaluated (https://clinicaltrials.gov/), as shown in Table 2 and Fig. 7. Future research can develop precise delivery systems and combination therapies to optimize therapeutic windows.

**Table 2:** Registered clinical trials of mitochondria-targeted therapeutics in cancer treatment

ClinicalTrials.gov ID	Phase	Start date (year)	Status	Targeted functions	Drugs used	Combination of drugs
NCT05680662	Early Phase 1	2023	Unknown status	ETC inhibitors	Metformin	Quercetin, EGCG, Zinc
NCT01981525	Phase 1	2014	Completed	ETC inhibitors	Metformin	
NCT03311308	Phase 1	2018	Recruiting	ETC inhibitors	Metformin	Pembrolizumab
NCT05136846	Phase 1	2021	Recruiting	ETC inhibitors	Papaverine	Carboplatin, Durvalumab
NCT05759312	Phase 1/2	2023	Unknown status	ETC inhibitors	Metformin	Zimberelimab
NCT03709147	Phase 2	2018	Unknown status	ETC inhibitors	Metformin	Metformin plus/minus Cyclic Fasting Mimicking diet (FMD)
NCT02874430	Phase 2	2016	Unknown status	ETC inhibitors	Metformin	Doxycycline
NCT03675893	Phase 2	2018	Active, not recruiting	ETC inhibitors	Metformin	Abemaciclib + Letrozole +/ − Metformin or Zotatifin
NCT04114136	Phase 2	2020	Recruiting	ETC inhibitors	Metformin	Nivolumab or Pembrolizumab
NCT04945148	Phase 2	2024	Recruiting	ETC inhibitors	Metformin	Temozolomide
NCT03664115	Phase 2	2018	Unknown status	ETC inhibitors	Itraconazole	Chemotherapy
NCT02168140	Phase 1	2014	Completed	TCA cycle	CPI-613	Bendamustine Hydrochloride
NCT05325281	Phase 1	2022	Recruiting	TCA cycle	CPI-613	Gemcitabine/Intensity-modulated Radiation Therapy
NCT02232152	Phase 1	2015	Completed	TCA cycle	CPI-613	Fluorouracil
NCT05070104	Phase 1	2023	Withdrawn	TCA cycle	CPI-613	Modified FOLFIRINOX + Bevacizumab
NCT02484391	Phase 1	2015	Completed	TCA cycle	CPI-613	Cytarabine
NCT04732065	Phase 1	2021	Recruiting	TCA cycle	ONC206	
NCT02806817	Early Phase 1	2016	Completed	TCA cycle	ME-344	Bevacizumab
NCT06611839	Phase 1/2	2024	Not yet recruiting	TCA cycle	Ivosidenib	Venetoclax
NCT03699319	Phase 1/2	2018	Completed	TCA cycle	CPI-613	mFOLFIRINOX
NCT02472626	Phase 1/2	2015	Withdrawn	TCA cycle	CPI-613	Cytarabine + Daunorubicin Hydrochloride
NCT03394027	Phase 2	2018	Completed	TCA cycle	ONC201	
NCT03374852	Phase 2	2017	Withdrawn	TCA cycle	CPI-613	Mfolfirnox
NCT02883036	Observational	2016	Unknown status	TCA cycle	Tigecycline	
NCT04109820	Not Applicable	2020	Recruiting	Mitochondrial-targeting antioxidant	MitoQ	
NCT05146843	Not Applicable	2021	Unknown status	Mitochondrial-targeting antioxidant	MitoQ	
NCT04827810	Phase 1	2021	Suspended	Hsp90 inhibitor	Gamitrinib	
NCT00773747	Phase 3	2008	Completed	Hsp90 inhibitor	Vorinostat (MK-0683)	Bortezomib
NCT05381909	Phase 1	2022	Active, not recruiting	Mitochondrial-derived activator of caspases	BGB-24714	Paclitaxel, Carboplatin
NCT04975204	Phase 1	2022	Unknown status	Mitochondrial-derived activator of caspases	TQB3909	
NCT05775575	Phase 1/2	2023	Unknown status	Mitochondrial-derived activator of caspases	TQB3909	
NCT00566410	Phase 1	2007	Completed	Pyruvate dehydrogenase kinase inhibitor	Dichloroacetate (DCA)	
NCT04122625	Phase 1/2	2019	Completed	Second mitochondrial activator of caspases (SMAC)	Debio 1143	Nivolumab
NCT03270176	Phase 1	2017	Completed	Second mitochondrial activator of caspases (SMAC)	Debio 1143	Avelumab
NCT02098161	Phase 2	2014	Completed	Second mitochondrial activator of caspases (SMAC)	LCL161	
NCT01955434	Phase 2	2013	Completed	Second mitochondrial activator of caspases (SMAC)	LCL161	LCL161 alone or with cyclophosphamide
NCT04651634	Phase 2	2021	Active, not recruiting	Reactive oxygen species scavenger	MIT-001	
NCT02071862	Phase 1	2014	Completed	Glutaminase inhibitor	CB-839	
NCT02520011	Phase 2	2016	Terminated	Mitochondrial calcium uniporter	Mitoxantrone	Cytarabine, Alvocidib
NCT01965834	Phase 2	2012	Terminated	Fatty acid oxidation	Fenofibrate	

## Future perspectives and challenges

Recent advances in mtDNA mutation profiling, mitochondrial transcriptomics, and nanotechnology-enabled targeted delivery have transformed mitochondrial-targeted therapies into critical frontiers in precision oncology, providing novel molecular foundations and technological support for personalized assessment and therapeutic strategies.^5,543^

### Personalized assessment value of mtDNA mutation profiling

mtDNA mutation screening offers crucial molecular insights into tumor mitochondrial functionality. Single-cell multi-omics analyses revealed significant dynamic heterogeneity in mtDNA mutation accumulation. Both germline genetic variations (such as mutations transmitted during fertilization) and somatic mutations continuously accumulate throughout the cell cycle.^544^ A comprehensive analysis of 206,663 immune cells further demonstrated substantial variations in mtDNA deletion purification mechanisms across different cellular subpopulations, suggesting cell type-dependent impacts of mitochondrial genome integrity on cellular fate.^545^ Notably, the clinical relevance of mtDNA mutations is tumor-type specificity. In colorectal cancer, mtDNA mutations are weakly associated with mitochondrial biogenesis or oxidative metabolism,^546^ whereas in early-stage lung adenocarcinoma, mtDNA mutations demonstrate exceptional diagnostic sensitivity in liquid biopsies.^547^ This contradiction underscores the necessity of functionally annotating mtDNA mutation profiles within the specific metabolic reprogramming context of individual tumor types.

### Multidimensional mitochondrial transcriptomics and proteomics

Innovative mitochondrial transcriptomics technologies have significantly enhanced spatiotemporal resolution in tumor metabolic assessment. The Mitochondrial Alteration Enrichment from Single-cell Transcriptomes to Establish Relatedness (MAESTER) technique improved mtDNA mutation detection sensitivity by 50-fold at the single-cell level,^548^ whereas capture-based mtRNA sequencing overcame detection limitations for low-expression tRNA transcripts.^549^ In pancreatic cancer, a 12-gene prognostic model based on nuclear-mitochondrial genes (NMGs) not only predicts immune microenvironment characteristics but also provides molecular guidance for personalized therapeutic strategies.^550^ Mitochondrial proteomics research has further elucidated critical mechanisms of tumor drug resistance. Stable isotope labeling techniques identified mitochondrial protein networks associated with ovarian cancer drug resistance,^551^ whereas hepatocellular carcinoma subproteomic analyses revealed potential therapeutic targets.^552^ The current challenge lies in integrating multi-omics data to establish a cross-scale mitochondrial functional assessment framework spanning genomics, transcriptomics, and proteomics.

### Nanomedicine delivery systems

Multiple functional nanocarriers have made significant advances in targeted delivery but face dual challenges in clinical translation. The long-term stability of membrane-coated nanoparticles remains a critical concern. Novel preservation strategies such as phospholipid bilayer modifications can improve functional integrity, but scalable production encounters technical bottlenecks.^553^ Targeting efficiency presents another challenge. While mitochondrial-targeting motifs such as TPP can increase delivery efficiency,^554^ off-target effects from nonspecific uptake necessitate optimization through dual-targeting strategies, such as hybrid nanoparticle-hydrogel systems.^555^ Recent research has demonstrated promising approaches, such as encapsulating 2-aminothiophene derivatives in calcium carbonate microparticles to reduce systemic toxicity,^556^ although potential immunogenicity risks associated with nanocarriers warrant careful consideration.

### Circulating cell-free mtDNA and genome editing

Circulating cell-free mtDNA (ccf-mtDNA) has demonstrated significant clinical value as a dynamic monitoring biomarker in solid tumors such as hepatocellular and renal carcinomas.^557^ A notable recent study by Liu et al. utilized capture-based mtDNA sequencing to analyze plasma samples from 1,168 participants, successfully constructing random forest algorithms and LASSO-Cox regression models for hepatocellular carcinoma detection and prognostic prediction.^558^ In the genome editing domain, mitochondrial-targeted meganuclease (mitoARCUS) and DdCBE technologies have shown improved mtDNA editing efficiency compared with traditional CRISPR, although tissue-specific delivery systems require further optimization.^559,560^ Three-dimensional tumor organoid models have emerged as promising platforms for evaluating individual variations in mitochondrial-targeted therapies, as their ability to preserve TME heterogeneity significantly enhances preclinical research predictive validity.^561^

### Future perspectives: translational pathways in precision medicine

Current research delineates three primary translational pathways for mitochondrion-targeted therapies. First, multimodal assessment systems integrating mtDNA mutation profiling, transcriptomics, and proteomics were established, as demonstrated in early diagnostic models for lung adenocarcinoma and prognostic NMG models for pancreatic cancer.^547,550^ Second, intelligent nanocarriers with spatiotemporal controllability could be combined with immune checkpoint inhibitors to generate synergistic therapeutic effects.^562^ Third, ccf-mtDNA can be utilized for dynamic treatment response monitoring and individualized dose adjustments through machine learning models.^563^ Critically, implementing personalized mitochondrial-targeted therapies requires transcending traditional “single-target” paradigms, establishing a multidimensional evaluation framework encompassing genomic stability, metabolic plasticity, and microenvironment adaptability.

## Conclusion

Mitochondria represent the core of complex biological processes controlling cancer development, progression, and therapeutic resistance. Their multifaceted roles in metabolic reprogramming, stress responses, signal transduction, and intercellular communication establish them as critical modulators in tumor biology and primary intervention targets. Advances in understanding mitochondrial function not only reveal the intricate complexity of these organelles but also highlight their potential as pivotal conduits for innovative cancer therapies. Despite significant breakthroughs, translating mitochondrial-targeted strategies into clinical success remains challenging. The pronounced heterogeneity of mitochondrial behavior across different tumor types underscores the necessity for precision approaches. Moreover, dynamic interactions between cancer cells and the TME, encompassing stromal components, immune cells, and hypoxic conditions, further complicate therapeutic targeting. The off-target effects of mitochondrial-targeted drugs necessitate refined delivery mechanisms and identification of tumor-specific mitochondrial vulnerabilities.

Future research should prioritize a deeper comprehension of tumor-specific mitochondrial characteristics and vulnerabilities. Elucidating variations in mitochondrial dynamics, bioenergetics, and signaling pathways across diverse tumor types will provide critical insights for developing effective personalized predictive tools and therapeutic strategies. Furthermore, optimizing delivery mechanisms to achieve precise mitochondrial targeting and integrating these therapies into combinatorial protocols can increase efficacy while minimizing adverse reactions. Critically, preclinical and clinical investigations should also address potential adaptive resistance mechanisms inherent in mitochondrial-targeted approaches.

The field of cancer mitochondrial research stands at an exhilarating juncture, offering unprecedented opportunities for oncological transformation. By leveraging the unique properties of mitochondria, researchers and clinicians can confront long-standing challenges in cancer treatment, including therapeutic resistance and immune evasion. Continued exploration of mitochondrial metabolism and its therapeutic effects promises to inspire a new era of precision oncology. This paradigm shift holds the potential to significantly improve patient outcomes and propel global efforts in combating cancer.
